# Genetic Variation of Goat Interferon Regulatory Factor 3 Gene and Its Implication in Goat Evolution

**DOI:** 10.1371/journal.pone.0161962

**Published:** 2016-09-06

**Authors:** Moses Okpeku, Ali Esmailizadeh, Adeniyi C. Adeola, Liping Shu, Yesheng Zhang, Yangzi Wang, Timothy M. Sanni, Ikhide G. Imumorin, Sunday O. Peters, Jiajin Zhang, Yang Dong, Wen Wang

**Affiliations:** 1 State Key Laboratory of Genetic Resources and Evolution, Kunming Institute of Zoology, Chinese Academy of Sciences (CAS), Kunming, Yunnan 650223, China; 2 Department of Animal Science, Niger Delta University, Wilberforce Island, Ammassoma, Bayelsa State, Nigeria; 3 School of Science and Information Engineering, Yunnan Agricultural University, Kunming 650201, China; 4 Laboratory of Applied Genomics and Synthetic Biology, College of Life Science, Kunming University of Science and Technology, Kunming 650500, China; 5 Department of Animal Breeding and Genetics, Federal University of Agriculture, Abeokuta, Ogun State, Nigeria; 6 Department of Animal Science, Shahid Bahonar University of Kerman, Kerman, PB 76169–133, Iran; 7 Animal Genetics and Genomics Laboratory, Office of International Programs, College of Agriculture and Life Sciences, Cornell University, Ithaca, USA; 8 Department of Animal Science, Berry College, Mount Berry, USA; Istituto di Genetica Molecolare, ITALY

## Abstract

The immune systems are fundamentally vital for evolution and survival of species; as such, selection patterns in innate immune loci are of special interest in molecular evolutionary research. The interferon regulatory factor (*IRF*) gene family control many different aspects of the innate and adaptive immune responses in vertebrates. Among these, *IRF3* is known to take active part in very many biological processes. We assembled and evaluated 1356 base pairs of the *IRF3* gene coding region in domesticated goats from Africa (Nigeria, Ethiopia and South Africa) and Asia (Iran and China) and the wild goat (*Capra aegagrus*). Five segregating sites with θ value of 0.0009 for this gene demonstrated a low diversity across the goats’ populations. Fu and Li tests were significantly positive but Tajima’s D test was significantly negative, suggesting its deviation from neutrality. Neighbor joining tree of *IRF3* gene in domesticated goats, wild goat and sheep showed that all domesticated goats have a closer relationship than with the wild goat and sheep. Maximum likelihood tree of the gene showed that different domesticated goats share a common ancestor and suggest single origin. Four unique haplotypes were observed across all the sequences, of which, one was particularly common to African goats (MOCH-K14-0425, Poitou and WAD). In assessing the evolution mode of the gene, we found that the codon model d_N_/d_S_ ratio for all goats was greater than one. Phylogenetic Analysis by Maximum Likelihood (PAML) gave a ω_0_ (d_N_/d_S_) value of 0.067 with LnL value of -6900.3 for the first Model (M1) while ω2 = 1.667 in model M2 with LnL value of -6900.3 with positive selection inferred in 3 codon sites. Mechanistic empirical combination (MEC) model for evaluating adaptive selection pressure on particular codons also confirmed adaptive selection pressure in three codons (207, 358 and 408) in *IRF3* gene. Positive diversifying selection inferred with recent evolutionary changes in domesticated goat *IRF3* led us to conclude that the gene evolution may have been influenced by domestication processes in goats.

## Introduction

Domesticated goats are very important livestock diversity; they play significant roles in the economy of many developing countries, particularly as source of organic protein in food, and savings for poor rural farmers. They are widely distributed across most continents of the world [1], over different ecological and geographic areas including humid tropical rain forest, hot desert regions, and cold to hypoxic high altitude regions, defying harsh environmental conditions and surviving under poor agrarian conditions [2].

Acquisition of new functions in genes is credited to adaptive selection pressures [3] in close association with phenotypes and fitness of organisms [4,5,6]. Adaptive selective pressure on genes has also been reported to be indicators of functional adaptations developed during the evolution of species that has the tendency of promoting species functional diversification [7].

The interferon regulatory factor (*IRF*) gene family control many different aspects of the innate and adaptive immune responses in vertebrates along with cells reactions to stress [8]. Approximately ten members of this gene family have been elucidated in many vertebrate species along with other related genes [9]. Among these, *IRF3* is known to play significant roles in many biological activities. First of all, *IRF3* serves as innate immune receptor activated upon recognition of specific pathogen-associated molecular patterns (PAMPs) [8, 10]. Secondly, it plays active role in many toll-like receptors (TLR) signaling pathways [11, 12, 13] and also influences many different cellular processes such as cell death and metabolism [14]. Its activities have also been associated with a number of health indices in humans, mice, sheep and cattle [15].

Selection patterns in innate immune loci are of special interest in molecular evolutionary research because immune systems are fundamentally vital for evolution and survival of species [16]. Understanding the evolutionary footprint of *IRF3* gene will therefore provide valuable information for reconstructing evolutionary history and adaptation process of the species, and may provide useful insights into the design of marker-assisted selection and breeding for genetic improvement in goats. In this study, we investigated the molecular evolutionary signatures that may exert selection processes in the *IRF3* gene in goats and identified evolution footprints that may influence adaptation to different environments.

## Materials and Methods

Complete protocols for genomic sequencing and assembly, scaffold anchorage and gene annotation for these genes are as published in an earlier work [17]. The sequences of 1356 base pairs encoding region of *IRF3* gene in domesticated goats from Nigeria (West Africa), Ethiopia (East Africa), South Africa (Southern part of Africa), Iran (West Asia) and China (East Asia) and wild goat (*Capra aegagrus*) were obtained from our goat resequencing data. *IRF3* coding sequences for Moroccan, Iranian goats and *Ovis aries* were obtained from NextGen Capra Project (http://52.193.26.230/view/ERP001579) and GenBank, respectively. Accession numbers of sequences downloaded from public databases and information on resequencing data are presented in Table 1.

**Table 1:** Accession Identification for goat *IRF3* Sequences downloaded from NCBI and NextGen Capra Project for Moroccan and Iranian goats.

Specie	Sample name	Location	Data Source	Accession Number
Goat	West African Dwarf (WAD)	Nigeria	Resequenced data	
	Red Sokoto	Nigeria	Resequenced data	
	Ethiopian_Borena	Ethiopia	Resequenced data	
	Ethiopia_Somali	Ethiopia	Resequenced data	
	Iran_Cashmere	Iran	Resequenced data	
	Iran meat goat	Iran	Resequenced data	
	South African Meat goat	South Africa	Resequenced data	
	South African poitou	South Africa	Resequenced data	
	Chinese Black Yunana (*Capra hircus*)	China	Resequenced data	
	Morocan goat (MOCH)	public databases	NextGen Capra Project	ERS421320 ERS154595 ERS154569 ERS154584
	Iranian goat (IRCH)	public databases	NCBI GenBank	ERS239011 ERS239007 ERS239027 ERS239043 ERS239030 ERS239028
Wild goat	*Capra aegagrus*	Iran	Resequenced data	
Sheep	*Ovis aries*	public databases	NCBI GenBank	DQ152970.1

Translated sequences were aligned in the MEGA software program (version 6.0) as published earlier [18]. The alignment was manually checked and corrected for any ambiguity. 42 sequences with the frame shift were removed because they are quite possibly from low quality sequencing. Finally 36 sequences remained (S1 Fig) and were used for further analyses. Gene tree was constructed by Neighbor-joining (NJ) method in MEGA 6.0 with bootstrap value set to 1000 and sheep (*Ovis aries)* as the out-group. Maximum likelihood (ML) tree was inferred using the PhyML program version 4.8 and rooted in *Ovis arie*, tree visualization and editing was done with MEGA.

To test the hypothesis of neutrality operating on the gene, DnaSP v5.10.01 [19] was used to estimate population statistics including number of segregation sites, haplotype diversity, Fu and Li’s and Tajima’s *D* statistics [20] in goat sequences alone.

Adaptive selection pressure was tested using tree topology branch lengths calculated by codeML model in PAML package version 4 [21]. The F3 × 4 codon frequency model calculated using the nucleotide frequencies at the three codon positions was used. To detect positive selection at individual codons within the gene pair of models were compared using codeML: M1 (neutral model) was compared with M2 (adaptive model) and M7 was compared against M8 model [22]. Statistically significant evidence of positive selection was inferred with likelihood ratio test (LRT).

The influence of positively diversifying selection on genes can be inferred when ratio (ω) of non-synonymous (d_N_) to synonymous (d_S_) substitution rates exceeds one. The value of *ω* serves as a measure of comparative evolutionary patterns of codons and lineages [23]. To further confirm codon site selection pressure, multiple codon sequence alignments of *IRF3* for goats alone were submitted to the Selecton Server, version 2.2 (http://selecton.tau.ac.il/). Selecton version 2.2 allows the ω ratio to shift among codons within the multiple sequence alignments (MSA) and this parameter is estimated by maximum-likelihood value via Bayesian inference approach [24]. Additionally, the results from Selecton version 2.2 are visualized with color scales representing the different types of selection.

## Results

**Fig 1:**
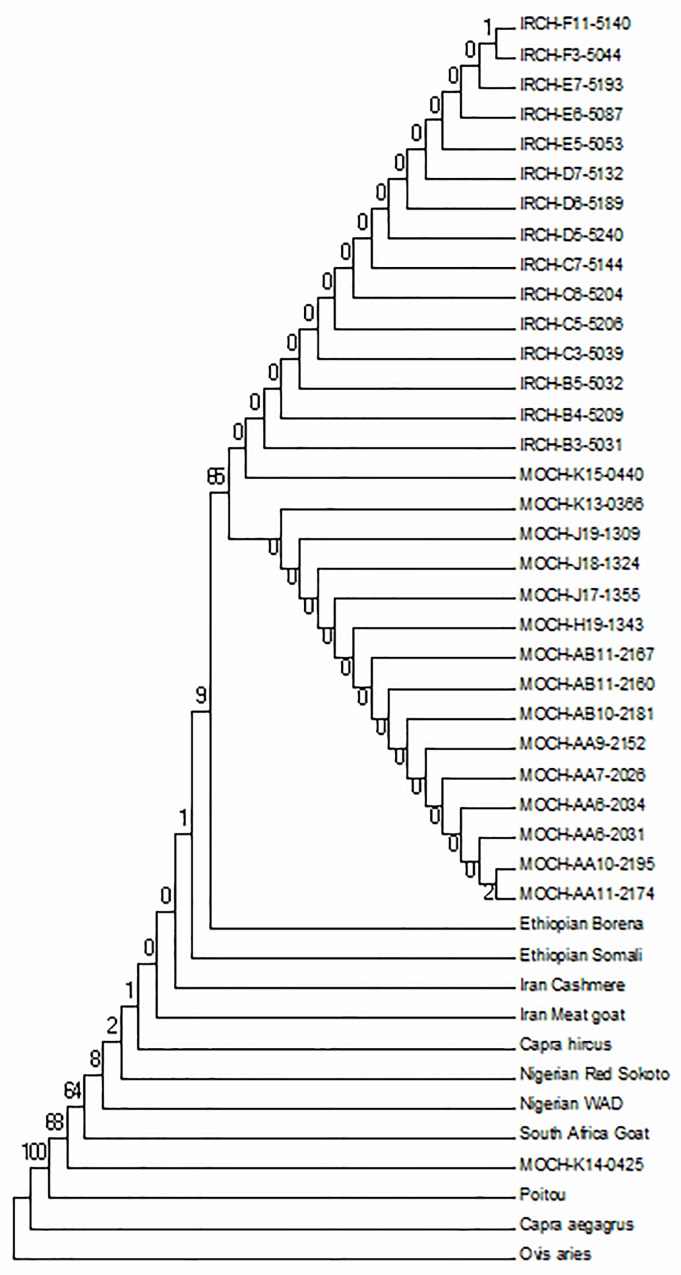
Neighbor joining phylogenetic trees for goat *IRF3* gene sequences constructed by Neighbor-joining (NJ) method in MEGA 6.0 with bootstrap value set to 1000 and *Ovis aries IRF3* gene sequence as outgroup. MOCH = Moroccan goats; IRCH = Iranian goats; WAD = West African Dwarf.

**Fig 2:**
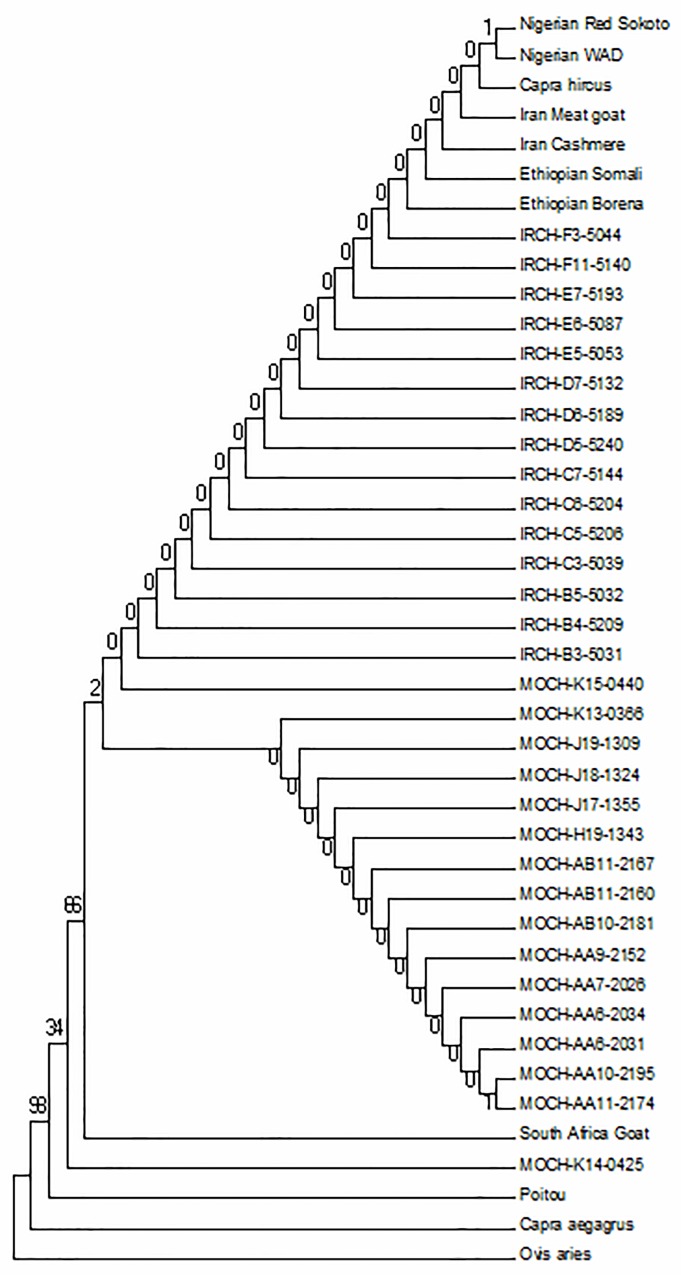
The phylogeny of goat *IRF3* gene was inferred by the maximum likelihood (ML) method using the PhyML program in MEGA software to analyze aligned sequences and this tree was rooted using the *Ovis aries IRF3* gene sequence. MOCH = Moroccan goats; IRCH = Iranian goats; WAD = West African Dwarf.

To understand the pattern of evolution of the *IRF3* gene in goats, we assembled *IRF3* gene coding sequences in domesticated and wild goats from our resequencing data and we also used the available sequences for the gene in public databases. To understand the basis for evolutionary patterns in the gene in the domesticated breeds and wild goat, we conducted phylogenetic analysis of aligned gene sequences in MEGA. Gene trees were constructed by Neighbor-joining (NJ) method in MEGA 6.0 with bootstrap value set to 1000 and *Ovis aries* as the out-group (Fig 1). Maximum likelihood (ML) tree was inferred using the PhyML program version 4.8 and rooted in *Ovis arie* (Fig 2). The gene trees showed closer relationships among all domesticated goats than with the wild goat and sheep *IRF3* gene. ML tree showed that the *IRF3* genes in different domesticated goats share a common ancestor, as illustrated in the length of the tree branches, suggesting single origin of domesticated goats.

To assess if the evolution of *IRF3* gene in goats deviates from neutrality (Table 2), we tested the hypothesis of neutrality with DnaSP v5.10.01 and estimated population statistics including number of segregation sites, haplotype diversity, Fu and Li’s and Tajima’s *D* statistics in goat sequences alone. Five segregating sites were observed in the coding sequences of domesticated and wild goats. Tajima’s D was significantly negative (P < 0.05) while the Fu and Li’s tests were significantly positive (P < 0.05). Four unique haplotypes (Hap_1, Hap_2, Hap_3 and Hap_4) were observed across all the sequences (Table 3), of which Hap_2 was particularly common to African goats (MOCH-K14-0425, Poitou and WAD), Hap_1 found in all domesticated goats, Hap_3 in the wild goat and Hap_4 associated with sheep *Ovis aries*.

**Table 2:** Population statistics and neutrality test in *IRF3* gene in goats alone and goats with Sheep.

	Population statistics				Neutrality test			
Sequences	M	S	Ps	θ	Tajima’s *D*	P- Value	Fu & Li’s	P- Value
Goats only	41	5	0.004	0.0009	-1.807	0.035	1.543	0.025
Goats and sheep	42	36	0.218	0.006	-2.551	0.01	1.983	0.02

M = Number of sequences

S = Number of segregating sites

Ps = Population diversity

θ = Haplotype diversity.

**Table 3:** Haplotypes found in different goat types.

Haplotype ID	Haplotype	Population where haplotye is found
Hap_1	CGACGGTCAAC	All domesticated goats except MOCH-K14-0425, Poitou, WAD
Hap_2	CGACGGTCCAC	MOCH-K14-0425, Poitou, WAD
Hap_3	CGTGGGTCCGT	*Capra_aegagrus*
Hap_4	GTTGACCTCGT	*Ovis_aries*

Hap = Haplotype

MOCH = Moroccan goats

WAD = West African Dwarf.

For further assessment of the evolution mode at the codon level, the codon models of PAML were used to infer estimates of ω under a maximum likelihood framework for all goat codon sequences (Table 4). Analysis was conducted using M1 versus M2 and M7 versus M8 PAML models and LRT was determined by using the likelihood logs. Model M1 gave a ω_0_ (d_N_/d_S_) value of 0.067 with LnL value of -6900.3 while ω2 = 1.667 in model M2 with LnL value of -6900.3; model M2 was judged favorable for this analysis. Model M2 demonstrated that more than 88% of the gene was under purifying selection pressure, while about 11% was under neutral selection pressure and 1% under active positive selection pressure; suggesting a small fraction of sites are under positive or diversifying selection in the genes, which might have led to the deviation from neutrality of the gene’s evolution.

**Table 4:** Inference of positive selection in *IRF3* genes using two pairs of models in Phylogenetic Analysis by Maximum Likelihood (PAML).

Gene	Model	Parameter Estimates	*LnL*	*LRT*	Positive selection codon sites
IRF3	Model1	_*P0*_ = 0.888 *p*1 = 0.112	-6900.3	0	Not Allowed
		*ω*_0_ = 0.067 *ω*_1_ = 1.000			
	Model2	_*P1*_ = 0.888 *p*_2 =_ 0.111 *p*_3_ = 0.006	-6900.3		3
		*ω*_1_ = 0.086 *ω*_2_ = 1.667 *ω*_3_ = 1.667			
	Model7	*p* = 0.632 *q* = 3. 182	-6897.87	0.476	Not Allowed
	Model8	*p*_0_ = 0.886 *p* = 0.562 *q* = 3.778	-6897.87		3
		*p*_*1*_ = 0.010 *ω* = 1.777			

(ω) = ratio of nonsynonymous-to-synonymous substitutions, Purified selection (p0), neutral selection (p2), positive selection (p3), substitution ratio for all sites, p and q = β distribution parameters, LnL = log likelihood; LRT = likelihood ratio.

**Fig 3:**
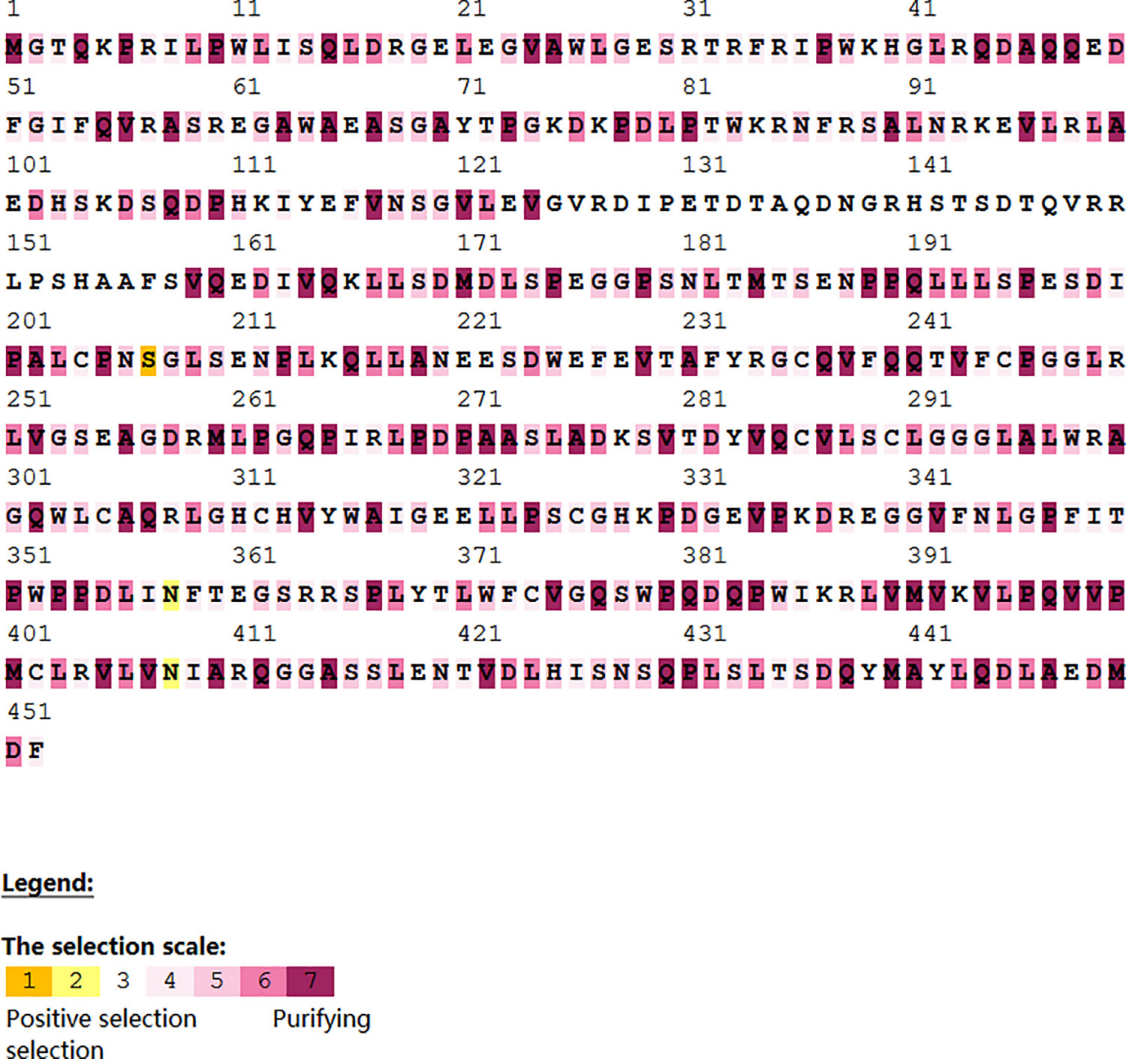
Selection pressures among goat *IRF3* gene sequences using mechanistic empirical combination (MEC) model of selecton online tool. Yellow and brown highlights represent positive selection, grey and white highlights represent neutral selection and purple highlight represent negative selection on codons.

Considering that PAML is prone to high false positive result, we also submitted aligned sequences of goats to the selecton online tool (http://selecton.tau.ac.il/) that employs the mechanistic empirical combination (MEC) model for evaluating adaptive selection pressure in codons. The MEC model takes into account the differences between amino acid replacement rates. Adaptive selection pressure was inferred in three codons (207, 358 and 408) in *IRF3* (Fig 3), identified under positive selection. A comparison of translated multiple sequence alignment of domesticated goats and wild goats in MEGA, revealed unique single nucleotide polymorphisms (SNPs) in the domestic goats in reference with the wild goat in the selected codons. Evolutionary changes in these codons resulted in non-synonymous changes in domesticated goats, which coded for different amino acid between domestic and wild goats (Table 5).

**Table 5:** Chromosome identity, number of exons and mutations in codons under positive selection pressure in *IRF3* gene.

Chromosome ID	No of Exons	Codon under selection	Mutation	Mutation Type	Domesticated goats Affected
18	8	207	W(TGG)→S(ACC)	NS	all domestic goats
		358	T(ACC)→N(AAC)	NS	MOCH-AA11-2174
					MOCH-AA6-2031
					MOCH-AA7-2034
					MOCH-AA9-2152
					MOCH-AB10-2181
					MOCH-H19-1343
					MOCH-J17-1355
					MOCH-J18-1324
					MOCH-H19-1309
					MOCH-K15-0440
					IRCH-B4-5209
					IRCH-B5-5032
					IRCH-C3-5039
					IRCH-C6-5204
					IRCH-C7-5144
					IRCH-D5-5240
					IRCH-D6-5189
					IRCH-E5-5053
					IRCH-E6-5087
					IRCH-E7-5193
					IRCH-F11-5140
					Ethiopia_Somali
					Iran_Cashmere
					Nigeria_Red_Sokoto
					Nigeria_WAD
			T(ACC)→Deletion	NS	MOCH-AB11-2160
					MOCH-AB11-2167
					MOCH-K13-0366
					IRCH-B3-5131
					IRCH-C5-5206
					IRCH-D7-5132
					IRCH-F3-5044
					Ethiopian_Borena
		408	D(GAC)→N(AAC)	NS	all domestic goats

MOCH = Moroccan goats

IRCH = Iranian goats

WAD = West African Dwarf

NS = Non-Synonimous.

## Discussion

Evolutionary studies in immune system genes have been widely conducted, especially in the toll like receptors (*TLR*) genes families such as the *IRF* gene family in many model species[15, 25, 26, 27]; however there is no published data on *IRF3* gene in goats. Our aim was to reveal evolutionary patterns and selection signatures in the goat innate immunity gene *IRF3*. Five segregating sites and θ value of 0.0009 for this gene demonstrated a low diversity across the goats’ populations, compared with 36 segregating sites when sheep *IRF3* sequence was added. This low diversity in goat population may not be unconnected with the number of samples used in the present study, data from a larger sample size may illuminate this farther. Neighbor Joining (NJ) and Maximum Likelihood (ML) gene trees showed close pattern of genetic relationship among domesticated goat, and a clear divergence between domesticated and wild goat types. Goats used in this study were sampled from diverse ecological environments across Asia and Africa, which may account for the genetic variation observed in the study. Four different haplotypes were observed in the study. One was found to be common in all goats, one only in wild goat and one particular haplotype was found only in three African domesticated goats including a North African goat MOCH-K14-0425 and dwarf goats (Poitou and WAD) from South Africa and West Africa respectively. This particular haplotype may represent a conserved segment of the sequence that survives the descent of many generations of reproduction [28], which may account for unique regional variation [29]. Also it is perhaps the product of environmental influence and local adaptation to environmental differences peculiar to some African goats. The dwarf goat breeds are particularly known to habit forest regions, they are hardy and often shown tendency of going feral. Strong humoral and innate immune responses have also been reported in these breeds [30]

Fu and Li’s tests were significant and positive while Tajima’s D test was significant but negative, indicating a deviation from neutrality and suggesting positive selection in the gene [31]. The influence of positive diversifying selection in genes can be inferred, when ratio of non-synonymous (d_N_) to synonymous (d_S_) substitution rates exceeds one [32]. Inferred d_N_/d_S_ ratio greater than one detected by codon models of PAML attests to the positive selection pressure in the *IRF3* gene. Although only about 1% of the codon sites in of gene were inferred to be under positive selection, this explains the observed deviation from neutrality of the gene’s evolution. Possible reason for evolution of adaptive selection in the gene might have resulted from adaptation to different environments. Goats, particularly the domesticated ones have always been influenced both by natural selection imposed by the environments and artificial selection influenced by human through selective breeding for specific production functions (such as milk, meat or both) [33]; a key factor in goat domestication. Breeding activities may have influence adaptive evolution in this gene.

Furthermore, mechanistic empirical combination (MEC) codon site selection also confirmed codons 207, 358 and 408 to be under strong adaptive selection pressure. Although all three codons were found in domesticated and wild goats, codons 207, 358 and 408 coded for Serine, Asparagine and Asparagine respectively in domesticated goats but Tryptophan, Tyrosine and Aspartic acid respectively in reference with the wild goat. Evolutionary changes of the gene in domesticated goats appeared to be more recent than in the wild goat, which may be connected with common domestication processes in domesticated goats. These codons probably play major roles in adaptive immune response. Adaptive selection has been reported to occur when a new or previously rare mutation bestows fitness benefit on individuals. Positively selected gene regions influence protein coding, host defense against pathogens, reproduction, speciation and adaptation to a new environment [34, 35, 36]. Although *IRF3* may not be directly involved in pathogen recognition, it has been reported to be active in signaling platform for transcriptional activities and many pathways involving resistance to viral infection [13, 37, 38]; these evidences suggest that the activation of these transcription factors is a precursor of other interferons and pathways implicated in adaptive immune responses intonation [37].

Remarkably, adaptive selection has been suggested to be connected with acquisition of new functions in genes [3] in connection with phenotypes and organism’s fitness [4, 5, 6]. This selective pressure has also been connected with functional adaptations gained in active evolution of species; which have the tendency of promoting functional diversification in species [7]. We therefore postulate that adaptive evolution observed in *IRF3* in domesticated goats is probably the result of the breeding processes associated with domestication.

*IRF3* gene has been reported to be involved in encoding proteins in connection with fundamental interactions between organisms and their environments [21] as such adaptive evolution of the gene may have taken place as a key factor in the evolution of goats for survival of unfriendly pathogenic environments during the process of domestication, when goats were bred for different functional purposes in various ecological environments. In support of this, a study based on *TLR2* genes published elsewhere [15], proposed that ruminant species are actively undergoing differential selective pressures. This process is attributed in part to direct selective breeding; resulting in population reduction and reduced effective population size of many ruminants species, which in turn may mirror initial domestication, breed formation or selection for specialized (e.g for meat, milk or fiber) [39]. An alternative proposal by Cui [40] is that breeding operations indirectly impacted changes in host-pathogen interactions suggesting that increased animal stocking density, pathogen transmission and load may also have increased selection for rapid adaptation in host and pathogen genes.

## Conclusion

*IRF3* gene have been widely studied in many species. However, to the best of our knowledge, the first report on adaptive selection of the gene in goats. The gene is showed low diversity in goats. Various analyses conducted in this study implicated positive or adaptive selection in the gene. Active adaptive evolution observed in *IRF3* gene suggested that the gene has experienced positive selection particularly in codons 207, 358 and 408. Our results suggest that adaptive evolution occurred in these codons in *IRF3* gene as a result of breeding processes associated with domestication and that the*se* genes may play important role in response to changing pathogenic pressure and adaptation. This gene may be promising target for further studies aiming at linking genetic variation to pathogen susceptibility in ruminants and other vertebrate groups that are threatened by emerging infectious diseases.

## Supporting Information

S1 Fig Aligned sequences of IRF3 gene used in the study. The gene is located on chromosome 18 and contains 8 exons.
(DOCX)
