# Supplementary material for: Genetic Variation of Goat Interferon Regulatory Factor 3 Gene and Its Implication in Goat Evolution
Source: PLoS One. 2016 Sep 6;11(9):e0161962. doi: 10.1371/journal.pone.0161962 (PMC5012607; doi:10.1371/journal.pone.0161962)
Supplement: S1 Fig — The gene is located on chromosome 18 and contains 8 exons. (DOCX) [file pone.0161962.s001.docx]

S1 Fig. Aligned sequences of IRF3 gene used in the study. The gene is located on chromosome 18 and contains 8 exons

>MOCH-AA10-2195

atgGGCACCCAGAAACCGCGTATTCTGCCGTGGCTGATTAGCCAGCTGGATCGTGGCGAACTGGAAGGCGTGGCGTGGCTGGGCGAAAGCCGTACCCGTTTTCGTATTCCGTGGAAACATGGCCTGCGTCAGGATGCGCAGCAGGAAGATTTTGGCATTTTTCAGGTGCGTGCGAGCCGTGAAGGCGCGTGGGCGGAAGCGAGCGGCGCGTATACCCCGGGCAAAGATAAACCGGATCTGCCGACCTGGAAACGTAACTTTCGTAGCGCGCTGAACCGTAAAGAAGTGCTGCGTCTGGCGGAAGATCATAGCAAAGATAGCCAGGATCCGCATAAAATTTATGAATTTGTGAACAGCGGCGTGCTGGAAGTGGGCGTGCGTGATATTCCGGAAACCGATACCGCGCAGGATAACGGCCGTCATAGCACCAGCGATACCCAGGTGCGTCGTCTGCCGAGCCATGCGGCGTTTAGCGTGCAGGAAGATATTGTGCAGAAACTGCTGAGCGATatgGATCTGAGCCCGGAAGGCGGCCCGAGCAACCTGACCatgACCAGCGAAAACCCGCCGCAGCTGCTGCTGAGCCCGGAAAGCGATATTCCGGCGCTGTGCCCGAACAGCGGCCTGAGCGAAAACCCGCTGAAACAGCTGCTGGCGAACGAAGAAAGCGATTGGGAATTTGAAGTGACCGCGTTTTATCGTGGCTGCCAGGTGTTTCAGCAGACCGTGTTTTGCCCGGGCGGCCTGCGTCTGGTGGGCAGCGAAGCGGGCGATCGTatgCTGCCGGGCCAGCCGATTCGTCTGCCGGATCCGGCGGCGAGCCTGGCGGATAAAAGCGTGACCGATTATGTGCAGTGCGTGCTGAGCTGCCTGGGCGGCGGCCTGGCGCTGTGGCGTGCGGGC---------------------CTGGGCCATTGCCATGTGTATTGGGCGATTGGCGAAGAACTGCTGCCGAGCTGCGGCCATAAACCGGATGGCGAAGTGCCGAAAGATCGTGAAGGCGGCGTGTTTAACCTGGGCCCGTTTATTACCCCGTGGCCGCCGGATCTGATTAACTTTACCGAAGGCAGCCGTCGTAGCCCGCTGTATACCCTGTGGTTTTGCGTGGGCCAGAGCTGGCCGCAGGATCAGCCGTGGATTAAACGTCTGGTGatgGTGAAAGTGCTGCCGCAGGTGGTGCCGatgTGCCTGCGTGTGCTGGTGAACATTGCGCGTCAGGGCGGCGCGAGCAGCCTGGAAAACACCGTGGATCTGCATATTAGCAACAGCCAGCCGCTGAGCCTGACCAGCGATCAGTATatgGCGTATCTGCAGGATCTGGCGGAAGATatgGATTTT

>MOCH-AA11-2174

atgGGCACCCAGAAACCGCGTATTCTGCCGTGGCTGATTAGCCAGCTGGATCGTGGCGAACTGGAAGGCGTGGCGTGGCTGGGCGAAAGCCGTACCCGTTTTCGTATTCCGTGGAAACATGGCCTGCGTCAGGATGCGCAGCAGGAAGATTTTGGCATTTTTCAGGTGCGTGCGAGCCGTGAAGGCGCGTGGGCGGAAGCGAGCGGCGCGTATACCCCGGGCAAAGATAAACCGGATCTGCCGACCTGGAAACGTAACTTTCGTAGCGCGCTGAACCGTAAAGAAGTGCTGCGTCTGGCGGAAGATCATAGCAAAGATAGCCAGGATCCGCATAAAATTTATGAATTTGTGAACAGCGGCGTGCTGGAAGTGGGCGTGCGTGATATTCCGGAAACCGATACCGCGCAGGATAACGGCCGTCATAGCACCAGCGATACCCAGGTGCGTCGTCTGCCGAGCCATGCGGCGTTTAGCGTGCAGGAAGATATTGTGCAGAAACTGCTGAGCGATatgGATCTGAGCCCGGAAGGCGGCCCGAGCAACCTGACCatgACCAGCGAAAACCCGCCGCAGCTGCTGCTGAGCCCGGAAAGCGATATTCCGGCGCTGTGCCCGAACAGCGGCCTGAGCGAAAACCCGCTGAAACAGCTGCTGGCGAACGAAGAAAGCGATTGGGAATTTGAAGTGACCGCGTTTTATCGTGGCTGCCAGGTGTTTCAGCAGACCGTGTTTTGCCCGGGCGGCCTGCGTCTGGTGGGCAGCGAAGCGGGCGATCGTatgCTGCCGGGCCAGCCGATTCGTCTGCCGGATCCGGCGGCGAGCCTGGCGGATAAAAGCGTGACCGATTATGTGCAGTGCGTGCTGAGCTGCCTGGGCGGCGGCCTGGCGCTGTGGCGTGCGGGC---------------------CTGGGCCATTGCCATGTGTATTGGGCGATTGGCGAAGAACTGCTGCCGAGCTGCGGCCATAAACCGGATGGCGAAGTGCCGAAAGATCGTGAAGGCGGCGTGTTTAACCTGGGCCCGTTTATTACCCCGTGGCCGCCGGATCTGATTAACTTTACCGAAGGCAGCCGTCGTAGCCCGCTGTATACCCTGTGGTTTTGCGTGGGCCAGAGCTGGCCGCAGGATCAGCCGTGGATTAAACGTCTGGTGatgGTGAAAGTGCTGCCGCAGGTGGTGCCGatgTGCCTGCGTGTGCTGGTGAACATTGCGCGTCAGGGCGGCGCGAGCAGCCTGGAAAACACCGTGGATCTGCATATTAGCAACAGCCAGCCGCTGAGCCTGACCAGCGATCAGTATatgGCGTATCTGCAGGATCTGGCGGAAGATatgGATTTT

>MOCH-AA6-2031

atgGGCACCCAGAAACCGCGTATTCTGCCGTGGCTGATTAGCCAGCTGGATCGTGGCGAACTGGAAGGCGTGGCGTGGCTGGGCGAAAGCCGTACCCGTTTTCGTATTCCGTGGAAACATGGCCTGCGTCAGGATGCGCAGCAGGAAGATTTTGGCATTTTTCAGGTGCGTGCGAGCCGTGAAGGCGCGTGGGCGGAAGCGAGCGGCGCGTATACCCCGGGCAAAGATAAACCGGATCTGCCGACCTGGAAACGTAACTTTCGTAGCGCGCTGAACCGTAAAGAAGTGCTGCGTCTGGCGGAAGATCATAGCAAAGATAGCCAGGATCCGCATAAAATTTATGAATTTGTGAACAGCGGCGTGCTGGAAGTGGGCGTGCGTGATATTCCGGAAACCGATACCGCGCAGGATAACGGCCGTCATAGCACCAGCGATACCCAGGTGCGTCGTCTGCCGAGCCATGCGGCGTTTAGCGTGCAGGAAGATATTGTGCAGAAACTGCTGAGCGATatgGATCTGAGCCCGGAAGGCGGCCCGAGCAACCTGACCatgACCAGCGAAAACCCGCCGCAGCTGCTGCTGAGCCCGGAAAGCGATATTCCGGCGCTGTGCCCGAACAGCGGCCTGAGCGAAAACCCGCTGAAACAGCTGCTGGCGAACGAAGAAAGCGATTGGGAATTTGAAGTGACCGCGTTTTATCGTGGCTGCCAGGTGTTTCAGCAGACCGTGTTTTGCCCGGGCGGCCTGCGTCTGGTGGGCAGCGAAGCGGGCGATCGTatgCTGCCGGGCCAGCCGATTCGTCTGCCGGATCCGGCGGCGAGCCTGGCGGATAAAAGCGTGACCGATTATGTGCAGTGCGTGCTGAGCTGCCTGGGCGGCGGCCTGGCGCTGTGGCGTGCGGGC---------------------CTGGGCCATTGCCATGTGTATTGGGCGATTGGCGAAGAACTGCTGCCGAGCTGCGGCCATAAACCGGATGGCGAAGTGCCGAAAGATCGTGAAGGCGGCGTGTTTAACCTGGGCCCGTTTATTACCCCGTGGCCGCCGGATCTGATTAACTTTACCGAAGGCAGCCGTCGTAGCCCGCTGTATACCCTGTGGTTTTGCGTGGGCCAGAGCTGGCCGCAGGATCAGCCGTGGATTAAACGTCTGGTGatgGTGAAAGTGCTGCCGCAGGTGGTGCCGatgTGCCTGCGTGTGCTGGTGAACATTGCGCGTCAGGGCGGCGCGAGCAGCCTGGAAAACACCGTGGATCTGCATATTAGCAACAGCCAGCCGCTGAGCCTGACCAGCGATCAGTATatgGCGTATCTGCAGGATCTGGCGGAAGATatgGATTTT

>MOCH-AA6-2034

atgGGCACCCAGAAACCGCGTATTCTGCCGTGGCTGATTAGCCAGCTGGATCGTGGCGAACTGGAAGGCGTGGCGTGGCTGGGCGAAAGCCGTACCCGTTTTCGTATTCCGTGGAAACATGGCCTGCGTCAGGATGCGCAGCAGGAAGATTTTGGCATTTTTCAGGTGCGTGCGAGCCGTGAAGGCGCGTGGGCGGAAGCGAGCGGCGCGTATACCCCGGGCAAAGATAAACCGGATCTGCCGACCTGGAAACGTAACTTTCGTAGCGCGCTGAACCGTAAAGAAGTGCTGCGTCTGGCGGAAGATCATAGCAAAGATAGCCAGGATCCGCATAAAATTTATGAATTTGTGAACAGCGGCGTGCTGGAAGTGGGCGTGCGTGATATTCCGGAAACCGATACCGCGCAGGATAACGGCCGTCATAGCACCAGCGATACCCAGGTGCGTCGTCTGCCGAGCCATGCGGCGTTTAGCGTGCAGGAAGATATTGTGCAGAAACTGCTGAGCGATatgGATCTGAGCCCGGAAGGCGGCCCGAGCAACCTGACCatgACCAGCGAAAACCCGCCGCAGCTGCTGCTGAGCCCGGAAAGCGATATTCCGGCGCTGTGCCCGAACAGCGGCCTGAGCGAAAACCCGCTGAAACAGCTGCTGGCGAACGAAGAAAGCGATTGGGAATTTGAAGTGACCGCGTTTTATCGTGGCTGCCAGGTGTTTCAGCAGACCGTGTTTTGCCCGGGCGGCCTGCGTCTGGTGGGCAGCGAAGCGGGCGATCGTatgCTGCCGGGCCAGCCGATTCGTCTGCCGGATCCGGCGGCGAGCCTGGCGGATAAAAGCGTGACCGATTATGTGCAGTGCGTGCTGAGCTGCCTGGGCGGCGGCCTGGCGCTGTGGCGTGCGGGC---------------------CTGGGCCATTGCCATGTGTATTGGGCGATTGGCGAAGAACTGCTGCCGAGCTGCGGCCATAAACCGGATGGCGAAGTGCCGAAAGATCGTGAAGGCGGCGTGTTTAACCTGGGCCCGTTTATTACCCCGTGGCCGCCGGATCTGATTAACTTTACCGAAGGCAGCCGTCGTAGCCCGCTGTATACCCTGTGGTTTTGCGTGGGCCAGAGCTGGCCGCAGGATCAGCCGTGGATTAAACGTCTGGTGatgGTGAAAGTGCTGCCGCAGGTGGTGCCGatgTGCCTGCGTGTGCTGGTGAACATTGCGCGTCAGGGCGGCGCGAGCAGCCTGGAAAACACCGTGGATCTGCATATTAGCAACAGCCAGCCGCTGAGCCTGACCAGCGATCAGTATatgGCGTATCTGCAGGATCTGGCGGAAGATatgGATTTT

>MOCH-AA7-2026

atgGGCACCCAGAAACCGCGTATTCTGCCGTGGCTGATTAGCCAGCTGGATCGTGGCGAACTGGAAGGCGTGGCGTGGCTGGGCGAAAGCCGTACCCGTTTTCGTATTCCGTGGAAACATGGCCTGCGTCAGGATGCGCAGCAGGAAGATTTTGGCATTTTTCAGGTGCGTGCGAGCCGTGAAGGCGCGTGGGCGGAAGCGAGCGGCGCGTATACCCCGGGCAAAGATAAACCGGATCTGCCGACCTGGAAACGTAACTTTCGTAGCGCGCTGAACCGTAAAGAAGTGCTGCGTCTGGCGGAAGATCATAGCAAAGATAGCCAGGATCCGCATAAAATTTATGAATTTGTGAACAGCGGCGTGCTGGAAGTGGGCGTGCGTGATATTCCGGAAACCGATACCGCGCAGGATAACGGCCGTCATAGCACCAGCGATACCCAGGTGCGTCGTCTGCCGAGCCATGCGGCGTTTAGCGTGCAGGAAGATATTGTGCAGAAACTGCTGAGCGATatgGATCTGAGCCCGGAAGGCGGCCCGAGCAACCTGACCatgACCAGCGAAAACCCGCCGCAGCTGCTGCTGAGCCCGGAAAGCGATATTCCGGCGCTGTGCCCGAACAGCGGCCTGAGCGAAAACCCGCTGAAACAGCTGCTGGCGAACGAAGAAAGCGATTGGGAATTTGAAGTGACCGCGTTTTATCGTGGCTGCCAGGTGTTTCAGCAGACCGTGTTTTGCCCGGGCGGCCTGCGTCTGGTGGGCAGCGAAGCGGGCGATCGTatgCTGCCGGGCCAGCCGATTCGTCTGCCGGATCCGGCGGCGAGCCTGGCGGATAAAAGCGTGACCGATTATGTGCAGTGCGTGCTGAGCTGCCTGGGCGGCGGCCTGGCGCTGTGGCGTGCGGGC---------------------CTGGGCCATTGCCATGTGTATTGGGCGATTGGCGAAGAACTGCTGCCGAGCTGCGGCCATAAACCGGATGGCGAAGTGCCGAAAGATCGTGAAGGCGGCGTGTTTAACCTGGGCCCGTTTATTACCCCGTGGCCGCCGGATCTGATTAACTTTACCGAAGGCAGCCGTCGTAGCCCGCTGTATACCCTGTGGTTTTGCGTGGGCCAGAGCTGGCCGCAGGATCAGCCGTGGATTAAACGTCTGGTGatgGTGAAAGTGCTGCCGCAGGTGGTGCCGatgTGCCTGCGTGTGCTGGTGAACATTGCGCGTCAGGGCGGCGCGAGCAGCCTGGAAAACACCGTGGATCTGCATATTAGCAACAGCCAGCCGCTGAGCCTGACCAGCGATCAGTATatgGCGTATCTGCAGGATCTGGCGGAAGATatgGATTTT

>MOCH-AA9-2152

atgGGCACCCAGAAACCGCGTATTCTGCCGTGGCTGATTAGCCAGCTGGATCGTGGCGAACTGGAAGGCGTGGCGTGGCTGGGCGAAAGCCGTACCCGTTTTCGTATTCCGTGGAAACATGGCCTGCGTCAGGATGCGCAGCAGGAAGATTTTGGCATTTTTCAGGTGCGTGCGAGCCGTGAAGGCGCGTGGGCGGAAGCGAGCGGCGCGTATACCCCGGGCAAAGATAAACCGGATCTGCCGACCTGGAAACGTAACTTTCGTAGCGCGCTGAACCGTAAAGAAGTGCTGCGTCTGGCGGAAGATCATAGCAAAGATAGCCAGGATCCGCATAAAATTTATGAATTTGTGAACAGCGGCGTGCTGGAAGTGGGCGTGCGTGATATTCCGGAAACCGATACCGCGCAGGATAACGGCCGTCATAGCACCAGCGATACCCAGGTGCGTCGTCTGCCGAGCCATGCGGCGTTTAGCGTGCAGGAAGATATTGTGCAGAAACTGCTGAGCGATatgGATCTGAGCCCGGAAGGCGGCCCGAGCAACCTGACCatgACCAGCGAAAACCCGCCGCAGCTGCTGCTGAGCCCGGAAAGCGATATTCCGGCGCTGTGCCCGAACAGCGGCCTGAGCGAAAACCCGCTGAAACAGCTGCTGGCGAACGAAGAAAGCGATTGGGAATTTGAAGTGACCGCGTTTTATCGTGGCTGCCAGGTGTTTCAGCAGACCGTGTTTTGCCCGGGCGGCCTGCGTCTGGTGGGCAGCGAAGCGGGCGATCGTatgCTGCCGGGCCAGCCGATTCGTCTGCCGGATCCGGCGGCGAGCCTGGCGGATAAAAGCGTGACCGATTATGTGCAGTGCGTGCTGAGCTGCCTGGGCGGCGGCCTGGCGCTGTGGCGTGCGGGC---------------------CTGGGCCATTGCCATGTGTATTGGGCGATTGGCGAAGAACTGCTGCCGAGCTGCGGCCATAAACCGGATGGCGAAGTGCCGAAAGATCGTGAAGGCGGCGTGTTTAACCTGGGCCCGTTTATTACCCCGTGGCCGCCGGATCTGATTAACTTTACCGAAGGCAGCCGTCGTAGCCCGCTGTATACCCTGTGGTTTTGCGTGGGCCAGAGCTGGCCGCAGGATCAGCCGTGGATTAAACGTCTGGTGatgGTGAAAGTGCTGCCGCAGGTGGTGCCGatgTGCCTGCGTGTGCTGGTGAACATTGCGCGTCAGGGCGGCGCGAGCAGCCTGGAAAACACCGTGGATCTGCATATTAGCAACAGCCAGCCGCTGAGCCTGACCAGCGATCAGTATatgGCGTATCTGCAGGATCTGGCGGAAGATatgGATTTT

>MOCH-AB10-2181

atgGGCACCCAGAAACCGCGTATTCTGCCGTGGCTGATTAGCCAGCTGGATCGTGGCGAACTGGAAGGCGTGGCGTGGCTGGGCGAAAGCCGTACCCGTTTTCGTATTCCGTGGAAACATGGCCTGCGTCAGGATGCGCAGCAGGAAGATTTTGGCATTTTTCAGGTGCGTGCGAGCCGTGAAGGCGCGTGGGCGGAAGCGAGCGGCGCGTATACCCCGGGCAAAGATAAACCGGATCTGCCGACCTGGAAACGTAACTTTCGTAGCGCGCTGAACCGTAAAGAAGTGCTGCGTCTGGCGGAAGATCATAGCAAAGATAGCCAGGATCCGCATAAAATTTATGAATTTGTGAACAGCGGCGTGCTGGAAGTGGGCGTGCGTGATATTCCGGAAACCGATACCGCGCAGGATAACGGCCGTCATAGCACCAGCGATACCCAGGTGCGTCGTCTGCCGAGCCATGCGGCGTTTAGCGTGCAGGAAGATATTGTGCAGAAACTGCTGAGCGATatgGATCTGAGCCCGGAAGGCGGCCCGAGCAACCTGACCatgACCAGCGAAAACCCGCCGCAGCTGCTGCTGAGCCCGGAAAGCGATATTCCGGCGCTGTGCCCGAACAGCGGCCTGAGCGAAAACCCGCTGAAACAGCTGCTGGCGAACGAAGAAAGCGATTGGGAATTTGAAGTGACCGCGTTTTATCGTGGCTGCCAGGTGTTTCAGCAGACCGTGTTTTGCCCGGGCGGCCTGCGTCTGGTGGGCAGCGAAGCGGGCGATCGTatgCTGCCGGGCCAGCCGATTCGTCTGCCGGATCCGGCGGCGAGCCTGGCGGATAAAAGCGTGACCGATTATGTGCAGTGCGTGCTGAGCTGCCTGGGCGGCGGCCTGGCGCTGTGGCGTGCGGGC---------------------CTGGGCCATTGCCATGTGTATTGGGCGATTGGCGAAGAACTGCTGCCGAGCTGCGGCCATAAACCGGATGGCGAAGTGCCGAAAGATCGTGAAGGCGGCGTGTTTAACCTGGGCCCGTTTATTACCCCGTGGCCGCCGGATCTGATTAACTTTACCGAAGGCAGCCGTCGTAGCCCGCTGTATACCCTGTGGTTTTGCGTGGGCCAGAGCTGGCCGCAGGATCAGCCGTGGATTAAACGTCTGGTGatgGTGAAAGTGCTGCCGCAGGTGGTGCCGatgTGCCTGCGTGTGCTGGTGAACATTGCGCGTCAGGGCGGCGCGAGCAGCCTGGAAAACACCGTGGATCTGCATATTAGCAACAGCCAGCCGCTGAGCCTGACCAGCGATCAGTATatgGCGTATCTGCAGGATCTGGCGGAAGATatgGATTTT

>MOCH-AB11-2160

atgGGCACCCAGAAACCGCGTATTCTGCCGTGGCTGATTAGCCAGCTGGATCGTGGCGAACTGGAAGGCGTGGCGTGGCTGGGCGAAAGCCGTACCCGTTTTCGTATTCCGTGGAAACATGGCCTGCGTCAGGATGCGCAGCAGGAAGATTTTGGCATTTTTCAGGTGCGTGCGAGCCGTGAAGGCGCGTGGGCGGAAGCGAGCGGCGCGTATACCCCGGGCAAAGATAAACCGGATCTGCCGACCTGGAAACGTAACTTTCGTAGCGCGCTGAACCGTAAAGAAGTGCTGCGTCTGGCGGAAGATCATAGCAAAGATAGCCAGGATCCGCATAAAATTTATGAATTTGTGAACAGCGGCGTGCTGGAAGTGGGCGTGCGTGATATTCCGGAAACCGATACCGCGCAGGATAACGGCCGTCATAGCACCAGCGATACCCAGGTGCGTCGTCTGCCGAGCCATGCGGCGTTTAGCGTGCAGGAAGATATTGTGCAGAAACTGCTGAGCGATatgGATCTGAGCCCGGAAGGCGGCCCGAGCAACCTGACCatgACCAGCGAAAACCCGCCGCAGCTGCTGCTGAGCCCGGAAAGCGATATTCCGGCGCTGTGCCCGAACAGCGGCCTGAGCGAAAACCCGCTGAAACAGCTGCTGGCGAACGAAGAAAGCGATTGGGAATTTGAAGTGACCGCGTTTTATCGTGGCTGCCAGGTGTTTCAGCAGACCGTGTTTTGCCCGGGCGGCCTGCGTCTGGTGGGCAGCGAAGCGGGCGATCGTatgCTGCCGGGCCAGCCGATTCGTCTGCCGGATCCGGCGGCGAGCCTGGCGGATAAAAGCGTGACCGATTATGTGCAGTGCGTGCTGAGCTGCCTGGGCGGCGGCCTGGCGCTGTGGCGTGCGGGC---------------------CTGGGCCATTGCCATGTGTATTGGGCGATTGGCGAAGAACTGCTGCCGAGCTGCGGCCATAAACCGGATGGCGAAGTGCCGAAAGATCGTGAAGGCGGCGTGTTTAACCTGGGCCCGTTTATTACCCCGTGGCCGCCGGATCTGATTAACTTTACCGAAGGCAGCCGTCGTAGCCCGCTGTATACCCTGTGGTTTTGCGTGGGCCAGAGCTGGCCGCAGGATCAGCCGTGGATTAAACGTCTGGTGatgGTGAAAGTGCTGCCGCAGGTGGTGCCGatgTGCCTGCGTGTGCTGGTGAACATTGCGCGTCAGGGCGGCGCGAGCAGCCTGGAAAACACCGTGGATCTGCATATTAGCAACAGCCAGCCGCTGAGCCTGACCAGCGATCAGTATatgGCGTATCTGCAGGATCTGGCGGAAGATatgGATTTT

>MOCH-AB11-2167

atgGGCACCCAGAAACCGCGTATTCTGCCGTGGCTGATTAGCCAGCTGGATCGTGGCGAACTGGAAGGCGTGGCGTGGCTGGGCGAAAGCCGTACCCGTTTTCGTATTCCGTGGAAACATGGCCTGCGTCAGGATGCGCAGCAGGAAGATTTTGGCATTTTTCAGGTGCGTGCGAGCCGTGAAGGCGCGTGGGCGGAAGCGAGCGGCGCGTATACCCCGGGCAAAGATAAACCGGATCTGCCGACCTGGAAACGTAACTTTCGTAGCGCGCTGAACCGTAAAGAAGTGCTGCGTCTGGCGGAAGATCATAGCAAAGATAGCCAGGATCCGCATAAAATTTATGAATTTGTGAACAGCGGCGTGCTGGAAGTGGGCGTGCGTGATATTCCGGAAACCGATACCGCGCAGGATAACGGCCGTCATAGCACCAGCGATACCCAGGTGCGTCGTCTGCCGAGCCATGCGGCGTTTAGCGTGCAGGAAGATATTGTGCAGAAACTGCTGAGCGATatgGATCTGAGCCCGGAAGGCGGCCCGAGCAACCTGACCatgACCAGCGAAAACCCGCCGCAGCTGCTGCTGAGCCCGGAAAGCGATATTCCGGCGCTGTGCCCGAACAGCGGCCTGAGCGAAAACCCGCTGAAACAGCTGCTGGCGAACGAAGAAAGCGATTGGGAATTTGAAGTGACCGCGTTTTATCGTGGCTGCCAGGTGTTTCAGCAGACCGTGTTTTGCCCGGGCGGCCTGCGTCTGGTGGGCAGCGAAGCGGGCGATCGTatgCTGCCGGGCCAGCCGATTCGTCTGCCGGATCCGGCGGCGAGCCTGGCGGATAAAAGCGTGACCGATTATGTGCAGTGCGTGCTGAGCTGCCTGGGCGGCGGCCTGGCGCTGTGGCGTGCGGGC---------------------CTGGGCCATTGCCATGTGTATTGGGCGATTGGCGAAGAACTGCTGCCGAGCTGCGGCCATAAACCGGATGGCGAAGTGCCGAAAGATCGTGAAGGCGGCGTGTTTAACCTGGGCCCGTTTATTACCCCGTGGCCGCCGGATCTGATTAACTTTACCGAAGGCAGCCGTCGTAGCCCGCTGTATACCCTGTGGTTTTGCGTGGGCCAGAGCTGGCCGCAGGATCAGCCGTGGATTAAACGTCTGGTGatgGTGAAAGTGCTGCCGCAGGTGGTGCCGatgTGCCTGCGTGTGCTGGTGAACATTGCGCGTCAGGGCGGCGCGAGCAGCCTGGAAAACACCGTGGATCTGCATATTAGCAACAGCCAGCCGCTGAGCCTGACCAGCGATCAGTATatgGCGTATCTGCAGGATCTGGCGGAAGATatgGATTTT

>MOCH-H19-1343

atgGGCACCCAGAAACCGCGTATTCTGCCGTGGCTGATTAGCCAGCTGGATCGTGGCGAACTGGAAGGCGTGGCGTGGCTGGGCGAAAGCCGTACCCGTTTTCGTATTCCGTGGAAACATGGCCTGCGTCAGGATGCGCAGCAGGAAGATTTTGGCATTTTTCAGGTGCGTGCGAGCCGTGAAGGCGCGTGGGCGGAAGCGAGCGGCGCGTATACCCCGGGCAAAGATAAACCGGATCTGCCGACCTGGAAACGTAACTTTCGTAGCGCGCTGAACCGTAAAGAAGTGCTGCGTCTGGCGGAAGATCATAGCAAAGATAGCCAGGATCCGCATAAAATTTATGAATTTGTGAACAGCGGCGTGCTGGAAGTGGGCGTGCGTGATATTCCGGAAACCGATACCGCGCAGGATAACGGCCGTCATAGCACCAGCGATACCCAGGTGCGTCGTCTGCCGAGCCATGCGGCGTTTAGCGTGCAGGAAGATATTGTGCAGAAACTGCTGAGCGATatgGATCTGAGCCCGGAAGGCGGCCCGAGCAACCTGACCatgACCAGCGAAAACCCGCCGCAGCTGCTGCTGAGCCCGGAAAGCGATATTCCGGCGCTGTGCCCGAACAGCGGCCTGAGCGAAAACCCGCTGAAACAGCTGCTGGCGAACGAAGAAAGCGATTGGGAATTTGAAGTGACCGCGTTTTATCGTGGCTGCCAGGTGTTTCAGCAGACCGTGTTTTGCCCGGGCGGCCTGCGTCTGGTGGGCAGCGAAGCGGGCGATCGTatgCTGCCGGGCCAGCCGATTCGTCTGCCGGATCCGGCGGCGAGCCTGGCGGATAAAAGCGTGACCGATTATGTGCAGTGCGTGCTGAGCTGCCTGGGCGGCGGCCTGGCGCTGTGGCGTGCGGGC---------------------CTGGGCCATTGCCATGTGTATTGGGCGATTGGCGAAGAACTGCTGCCGAGCTGCGGCCATAAACCGGATGGCGAAGTGCCGAAAGATCGTGAAGGCGGCGTGTTTAACCTGGGCCCGTTTATTACCCCGTGGCCGCCGGATCTGATTAACTTTACCGAAGGCAGCCGTCGTAGCCCGCTGTATACCCTGTGGTTTTGCGTGGGCCAGAGCTGGCCGCAGGATCAGCCGTGGATTAAACGTCTGGTGatgGTGAAAGTGCTGCCGCAGGTGGTGCCGatgTGCCTGCGTGTGCTGGTGAACATTGCGCGTCAGGGCGGCGCGAGCAGCCTGGAAAACACCGTGGATCTGCATATTAGCAACAGCCAGCCGCTGAGCCTGACCAGCGATCAGTATatgGCGTATCTGCAGGATCTGGCGGAAGATatgGATTTT

>MOCH-J17-1355

atgGGCACCCAGAAACCGCGTATTCTGCCGTGGCTGATTAGCCAGCTGGATCGTGGCGAACTGGAAGGCGTGGCGTGGCTGGGCGAAAGCCGTACCCGTTTTCGTATTCCGTGGAAACATGGCCTGCGTCAGGATGCGCAGCAGGAAGATTTTGGCATTTTTCAGGTGCGTGCGAGCCGTGAAGGCGCGTGGGCGGAAGCGAGCGGCGCGTATACCCCGGGCAAAGATAAACCGGATCTGCCGACCTGGAAACGTAACTTTCGTAGCGCGCTGAACCGTAAAGAAGTGCTGCGTCTGGCGGAAGATCATAGCAAAGATAGCCAGGATCCGCATAAAATTTATGAATTTGTGAACAGCGGCGTGCTGGAAGTGGGCGTGCGTGATATTCCGGAAACCGATACCGCGCAGGATAACGGCCGTCATAGCACCAGCGATACCCAGGTGCGTCGTCTGCCGAGCCATGCGGCGTTTAGCGTGCAGGAAGATATTGTGCAGAAACTGCTGAGCGATatgGATCTGAGCCCGGAAGGCGGCCCGAGCAACCTGACCatgACCAGCGAAAACCCGCCGCAGCTGCTGCTGAGCCCGGAAAGCGATATTCCGGCGCTGTGCCCGAACAGCGGCCTGAGCGAAAACCCGCTGAAACAGCTGCTGGCGAACGAAGAAAGCGATTGGGAATTTGAAGTGACCGCGTTTTATCGTGGCTGCCAGGTGTTTCAGCAGACCGTGTTTTGCCCGGGCGGCCTGCGTCTGGTGGGCAGCGAAGCGGGCGATCGTatgCTGCCGGGCCAGCCGATTCGTCTGCCGGATCCGGCGGCGAGCCTGGCGGATAAAAGCGTGACCGATTATGTGCAGTGCGTGCTGAGCTGCCTGGGCGGCGGCCTGGCGCTGTGGCGTGCGGGC---------------------CTGGGCCATTGCCATGTGTATTGGGCGATTGGCGAAGAACTGCTGCCGAGCTGCGGCCATAAACCGGATGGCGAAGTGCCGAAAGATCGTGAAGGCGGCGTGTTTAACCTGGGCCCGTTTATTACCCCGTGGCCGCCGGATCTGATTAACTTTACCGAAGGCAGCCGTCGTAGCCCGCTGTATACCCTGTGGTTTTGCGTGGGCCAGAGCTGGCCGCAGGATCAGCCGTGGATTAAACGTCTGGTGatgGTGAAAGTGCTGCCGCAGGTGGTGCCGatgTGCCTGCGTGTGCTGGTGAACATTGCGCGTCAGGGCGGCGCGAGCAGCCTGGAAAACACCGTGGATCTGCATATTAGCAACAGCCAGCCGCTGAGCCTGACCAGCGATCAGTATatgGCGTATCTGCAGGATCTGGCGGAAGATatgGATTTT

>MOCH-J18-1324

atgGGCACCCAGAAACCGCGTATTCTGCCGTGGCTGATTAGCCAGCTGGATCGTGGCGAACTGGAAGGCGTGGCGTGGCTGGGCGAAAGCCGTACCCGTTTTCGTATTCCGTGGAAACATGGCCTGCGTCAGGATGCGCAGCAGGAAGATTTTGGCATTTTTCAGGTGCGTGCGAGCCGTGAAGGCGCGTGGGCGGAAGCGAGCGGCGCGTATACCCCGGGCAAAGATAAACCGGATCTGCCGACCTGGAAACGTAACTTTCGTAGCGCGCTGAACCGTAAAGAAGTGCTGCGTCTGGCGGAAGATCATAGCAAAGATAGCCAGGATCCGCATAAAATTTATGAATTTGTGAACAGCGGCGTGCTGGAAGTGGGCGTGCGTGATATTCCGGAAACCGATACCGCGCAGGATAACGGCCGTCATAGCACCAGCGATACCCAGGTGCGTCGTCTGCCGAGCCATGCGGCGTTTAGCGTGCAGGAAGATATTGTGCAGAAACTGCTGAGCGATatgGATCTGAGCCCGGAAGGCGGCCCGAGCAACCTGACCatgACCAGCGAAAACCCGCCGCAGCTGCTGCTGAGCCCGGAAAGCGATATTCCGGCGCTGTGCCCGAACAGCGGCCTGAGCGAAAACCCGCTGAAACAGCTGCTGGCGAACGAAGAAAGCGATTGGGAATTTGAAGTGACCGCGTTTTATCGTGGCTGCCAGGTGTTTCAGCAGACCGTGTTTTGCCCGGGCGGCCTGCGTCTGGTGGGCAGCGAAGCGGGCGATCGTatgCTGCCGGGCCAGCCGATTCGTCTGCCGGATCCGGCGGCGAGCCTGGCGGATAAAAGCGTGACCGATTATGTGCAGTGCGTGCTGAGCTGCCTGGGCGGCGGCCTGGCGCTGTGGCGTGCGGGC---------------------CTGGGCCATTGCCATGTGTATTGGGCGATTGGCGAAGAACTGCTGCCGAGCTGCGGCCATAAACCGGATGGCGAAGTGCCGAAAGATCGTGAAGGCGGCGTGTTTAACCTGGGCCCGTTTATTACCCCGTGGCCGCCGGATCTGATTAACTTTACCGAAGGCAGCCGTCGTAGCCCGCTGTATACCCTGTGGTTTTGCGTGGGCCAGAGCTGGCCGCAGGATCAGCCGTGGATTAAACGTCTGGTGatgGTGAAAGTGCTGCCGCAGGTGGTGCCGatgTGCCTGCGTGTGCTGGTGAACATTGCGCGTCAGGGCGGCGCGAGCAGCCTGGAAAACACCGTGGATCTGCATATTAGCAACAGCCAGCCGCTGAGCCTGACCAGCGATCAGTATatgGCGTATCTGCAGGATCTGGCGGAAGATatgGATTTT

>MOCH-J19-1309

atgGGCACCCAGAAACCGCGTATTCTGCCGTGGCTGATTAGCCAGCTGGATCGTGGCGAACTGGAAGGCGTGGCGTGGCTGGGCGAAAGCCGTACCCGTTTTCGTATTCCGTGGAAACATGGCCTGCGTCAGGATGCGCAGCAGGAAGATTTTGGCATTTTTCAGGTGCGTGCGAGCCGTGAAGGCGCGTGGGCGGAAGCGAGCGGCGCGTATACCCCGGGCAAAGATAAACCGGATCTGCCGACCTGGAAACGTAACTTTCGTAGCGCGCTGAACCGTAAAGAAGTGCTGCGTCTGGCGGAAGATCATAGCAAAGATAGCCAGGATCCGCATAAAATTTATGAATTTGTGAACAGCGGCGTGCTGGAAGTGGGCGTGCGTGATATTCCGGAAACCGATACCGCGCAGGATAACGGCCGTCATAGCACCAGCGATACCCAGGTGCGTCGTCTGCCGAGCCATGCGGCGTTTAGCGTGCAGGAAGATATTGTGCAGAAACTGCTGAGCGATatgGATCTGAGCCCGGAAGGCGGCCCGAGCAACCTGACCatgACCAGCGAAAACCCGCCGCAGCTGCTGCTGAGCCCGGAAAGCGATATTCCGGCGCTGTGCCCGAACAGCGGCCTGAGCGAAAACCCGCTGAAACAGCTGCTGGCGAACGAAGAAAGCGATTGGGAATTTGAAGTGACCGCGTTTTATCGTGGCTGCCAGGTGTTTCAGCAGACCGTGTTTTGCCCGGGCGGCCTGCGTCTGGTGGGCAGCGAAGCGGGCGATCGTatgCTGCCGGGCCAGCCGATTCGTCTGCCGGATCCGGCGGCGAGCCTGGCGGATAAAAGCGTGACCGATTATGTGCAGTGCGTGCTGAGCTGCCTGGGCGGCGGCCTGGCGCTGTGGCGTGCGGGC---------------------CTGGGCCATTGCCATGTGTATTGGGCGATTGGCGAAGAACTGCTGCCGAGCTGCGGCCATAAACCGGATGGCGAAGTGCCGAAAGATCGTGAAGGCGGCGTGTTTAACCTGGGCCCGTTTATTACCCCGTGGCCGCCGGATCTGATTAACTTTACCGAAGGCAGCCGTCGTAGCCCGCTGTATACCCTGTGGTTTTGCGTGGGCCAGAGCTGGCCGCAGGATCAGCCGTGGATTAAACGTCTGGTGatgGTGAAAGTGCTGCCGCAGGTGGTGCCGatgTGCCTGCGTGTGCTGGTGAACATTGCGCGTCAGGGCGGCGCGAGCAGCCTGGAAAACACCGTGGATCTGCATATTAGCAACAGCCAGCCGCTGAGCCTGACCAGCGATCAGTATatgGCGTATCTGCAGGATCTGGCGGAAGATatgGATTTT

>MOCH-K13-0366

atgGGCACCCAGAAACCGCGTATTCTGCCGTGGCTGATTAGCCAGCTGGATCGTGGCGAACTGGAAGGCGTGGCGTGGCTGGGCGAAAGCCGTACCCGTTTTCGTATTCCGTGGAAACATGGCCTGCGTCAGGATGCGCAGCAGGAAGATTTTGGCATTTTTCAGGTGCGTGCGAGCCGTGAAGGCGCGTGGGCGGAAGCGAGCGGCGCGTATACCCCGGGCAAAGATAAACCGGATCTGCCGACCTGGAAACGTAACTTTCGTAGCGCGCTGAACCGTAAAGAAGTGCTGCGTCTGGCGGAAGATCATAGCAAAGATAGCCAGGATCCGCATAAAATTTATGAATTTGTGAACAGCGGCGTGCTGGAAGTGGGCGTGCGTGATATTCCGGAAACCGATACCGCGCAGGATAACGGCCGTCATAGCACCAGCGATACCCAGGTGCGTCGTCTGCCGAGCCATGCGGCGTTTAGCGTGCAGGAAGATATTGTGCAGAAACTGCTGAGCGATatgGATCTGAGCCCGGAAGGCGGCCCGAGCAACCTGACCatgACCAGCGAAAACCCGCCGCAGCTGCTGCTGAGCCCGGAAAGCGATATTCCGGCGCTGTGCCCGAACAGCGGCCTGAGCGAAAACCCGCTGAAACAGCTGCTGGCGAACGAAGAAAGCGATTGGGAATTTGAAGTGACCGCGTTTTATCGTGGCTGCCAGGTGTTTCAGCAGACCGTGTTTTGCCCGGGCGGCCTGCGTCTGGTGGGCAGCGAAGCGGGCGATCGTatgCTGCCGGGCCAGCCGATTCGTCTGCCGGATCCGGCGGCGAGCCTGGCGGATAAAAGCGTGACCGATTATGTGCAGTGCGTGCTGAGCTGCCTGGGCGGCGGCCTGGCGCTGTGGCGTGCGGGC---------------------CTGGGCCATTGCCATGTGTATTGGGCGATTGGCGAAGAACTGCTGCCGAGCTGCGGCCATAAACCGGATGGCGAAGTGCCGAAAGATCGTGAAGGCGGCGTGTTTAACCTGGGCCCGTTTATTACCCCGTGGCCGCCGGATCTGATTAACTTTACCGAAGGCAGCCGTCGTAGCCCGCTGTATACCCTGTGGTTTTGCGTGGGCCAGAGCTGGCCGCAGGATCAGCCGTGGATTAAACGTCTGGTGatgGTGAAAGTGCTGCCGCAGGTGGTGCCGatgTGCCTGCGTGTGCTGGTGAACATTGCGCGTCAGGGCGGCGCGAGCAGCCTGGAAAACACCGTGGATCTGCATATTAGCAACAGCCAGCCGCTGAGCCTGACCAGCGATCAGTATatgGCGTATCTGCAGGATCTGGCGGAAGATatgGATTTT

>MOCH-K14-0425

atgGGCACCCAGAAACCGCGTATTCTGCCGTGGCTGATTAGCCAGCTGGATCGTGGCGAACTGGAAGGCGTGGCGTGGCTGGGCGAAAGCCGTACCCGTTTTCGTATTCCGTGGAAACATGGCCTGCGTCAGGATGCGCAGCAGGAAGATTTTGGCATTTTTCAGGTGCGTGCGAGCCGTGAAGGCGCGTGGGCGGAAGCGAGCGGCGCGTATACCCCGGGCAAAGATAAACCGGATCTGCCGACCTGGAAACGTAACTTTCGTAGCGCGCTGAACCGTAAAGAAGTGCTGCGTCTGGCGGAAGATCATAGCAAAGATAGCCAGGATCCGCATAAAATTTATGAATTTGTGAACAGCGGCGTGCTGGAAGTGGGCGTGCGTGATATTCCGGAAACCGATACCGCGCAGGATAACGGCCGTCATAGCACCAGCGATACCCAGGTGCGTCGTCTGCCGAGCCATGCGGCGTTTAGCGTGCAGGAAGATATTGTGCAGAAACTGCTGAGCGATatgGATCTGAGCCCGGAAGGCGGCCCGAGCAACCTGACCatgACCAGCGAAAACCCGCCGCAGCTGCTGCTGAGCCCGGAAAGCGATATTCCGGCGCTGTGCCCGAACAGCGGCCTGAGCGAAAACCCGCTGAAACAGCTGCTGGCGAACGAAGAAAGCGATTGGGAATTTGAAGTGACCGCGTTTTATCGTGGCTGCCAGGTGTTTCAGCAGACCGTGTTTTGCCCGGGCGGCCTGCGTCTGGTGGGCAGCGAAGCGGGCGATCGTatgCTGCCGGGCCAGCCGATTCGTCTGCCGGATCCGGCGGCGAGCCTGGCGGATAAAAGCGTGACCGATTATGTGCAGTGCGTGCTGAGCTGCCTGGGCGGCGGCCTGGCGCTGTGGCGTGCGGGC---------------------CTGGGCCATTGCCATGTGTATTGGGCGATTGGCGAAGAACTGCTGCCGAGCTGCGGCCATAAACCGGATGGCGAAGTGCCGAAAGATCGTGAAGGCGGCGTGTTTAACCTGGGCCCGTTTATTACCCCGTGGCCGCCGGATCTGATTACCTTTACCGAAGGCAGCCGTCGTAGCCCGCTGTATACCCTGTGGTTTTGCGTGGGCCAGAGCTGGCCGCAGGATCAGCCGTGGATTAAACGTCTGGTGatgGTGAAAGTGCTGCCGCAGGTGGTGCCGatgTGCCTGCGTGTGCTGGTGAACATTGCGCGTCAGGGCGGCGCGAGCAGCCTGGAAAACACCGTGGATCTGCATATTAGCAACAGCCAGCCGCTGAGCCTGACCAGCGATCAGTATatgGCGTATCTGCAGGATCTGGCGGAAGATatgGATTTT

>MOCH-K15-0440

atgGGCACCCAGAAACCGCGTATTCTGCCGTGGCTGATTAGCCAGCTGGATCGTGGCGAACTGGAAGGCGTGGCGTGGCTGGGCGAAAGCCGTACCCGTTTTCGTATTCCGTGGAAACATGGCCTGCGTCAGGATGCGCAGCAGGAAGATTTTGGCATTTTTCAGGTGCGTGCGAGCCGTGAAGGCGCGTGGGCGGAAGCGAGCGGCGCGTATACCCCGGGCAAAGATAAACCGGATCTGCCGACCTGGAAACGTAACTTTCGTAGCGCGCTGAACCGTAAAGAAGTGCTGCGTCTGGCGGAAGATCATAGCAAAGATAGCCAGGATCCGCATAAAATTTATGAATTTGTGAACAGCGGCGTGCTGGAAGTGGGCGTGCGTGATATTCCGGAAACCGATACCGCGCAGGATAACGGCCGTCATAGCACCAGCGATACCCAGGTGCGTCGTCTGCCGAGCCATGCGGCGTTTAGCGTGCAGGAAGATATTGTGCAGAAACTGCTGAGCGATatgGATCTGAGCCCGGAAGGCGGCCCGAGCAACCTGACCatgACCAGCGAAAACCCGCCGCAGCTGCTGCTGAGCCCGGAAAGCGATATTCCGGCGCTGTGCCCGAACAGCGGCCTGAGCGAAAACCCGCTGAAACAGCTGCTGGCGAACGAAGAAAGCGATTGGGAATTTGAAGTGACCGCGTTTTATCGTGGCTGCCAGGTGTTTCAGCAGACCGTGTTTTGCCCGGGCGGCCTGCGTCTGGTGGGCAGCGAAGCGGGCGATCGTatgCTGCCGGGCCAGCCGATTCGTCTGCCGGATCCGGCGGCGAGCCTGGCGGATAAAAGCGTGACCGATTATGTGCAGTGCGTGCTGAGCTGCCTGGGCGGCGGCCTGGCGCTGTGGCGTGCGGGC---------------------CTGGGCCATTGCCATGTGTATTGGGCGATTGGCGAAGAACTGCTGCCGAGCTGCGGCCATAAACCGGATGGCGAAGTGCCGAAAGATCGTGAAGGCGGCGTGTTTAACCTGGGCCCGTTTATTACCCCGTGGCCGCCGGATCTGATTAACTTTACCGAAGGCAGCCGTCGTAGCCCGCTGTATACCCTGTGGTTTTGCGTGGGCCAGAGCTGGCCGCAGGATCAGCCGTGGATTAAACGTCTGGTGatgGTGAAAGTGCTGCCGCAGGTGGTGCCGatgTGCCTGCGTGTGCTGGTGAACATTGCGCGTCAGGGCGGCGCGAGCAGCCTGGAAAACACCGTGGATCTGCATATTAGCAACAGCCAGCCGCTGAGCCTGACCAGCGATCAGTATatgGCGTATCTGCAGGATCTGGCGGAAGATatgGATTTT

>IRCH-B3-5031

atgGGCACCCAGAAACCGCGTATTCTGCCGTGGCTGATTAGCCAGCTGGATCGTGGCGAACTGGAAGGCGTGGCGTGGCTGGGCGAAAGCCGTACCCGTTTTCGTATTCCGTGGAAACATGGCCTGCGTCAGGATGCGCAGCAGGAAGATTTTGGCATTTTTCAGGTGCGTGCGAGCCGTGAAGGCGCGTGGGCGGAAGCGAGCGGCGCGTATACCCCGGGCAAAGATAAACCGGATCTGCCGACCTGGAAACGTAACTTTCGTAGCGCGCTGAACCGTAAAGAAGTGCTGCGTCTGGCGGAAGATCATAGCAAAGATAGCCAGGATCCGCATAAAATTTATGAATTTGTGAACAGCGGCGTGCTGGAAGTGGGCGTGCGTGATATTCCGGAAACCGATACCGCGCAGGATAACGGCCGTCATAGCACCAGCGATACCCAGGTGCGTCGTCTGCCGAGCCATGCGGCGTTTAGCGTGCAGGAAGATATTGTGCAGAAACTGCTGAGCGATatgGATCTGAGCCCGGAAGGCGGCCCGAGCAACCTGACCatgACCAGCGAAAACCCGCCGCAGCTGCTGCTGAGCCCGGAAAGCGATATTCCGGCGCTGTGCCCGAACAGCGGCCTGAGCGAAAACCCGCTGAAACAGCTGCTGGCGAACGAAGAAAGCGATTGGGAATTTGAAGTGACCGCGTTTTATCGTGGCTGCCAGGTGTTTCAGCAGACCGTGTTTTGCCCGGGCGGCCTGCGTCTGGTGGGCAGCGAAGCGGGCGATCGTatgCTGCCGGGCCAGCCGATTCGTCTGCCGGATCCGGCGGCGAGCCTGGCGGATAAAAGCGTGACCGATTATGTGCAGTGCGTGCTGAGCTGCCTGGGCGGCGGCCTGGCGCTGTGGCGTGCGGGC---------------------CTGGGCCATTGCCATGTGTATTGGGCGATTGGCGAAGAACTGCTGCCGAGCTGCGGCCATAAACCGGATGGCGAAGTGCCGAAAGATCGTGAAGGCGGCGTGTTTAACCTGGGCCCGTTTATTACCCCGTGGCCGCCGGATCTGATTAACTTTACCGAAGGCAGCCGTCGTAGCCCGCTGTATACCCTGTGGTTTTGCGTGGGCCAGAGCTGGCCGCAGGATCAGCCGTGGATTAAACGTCTGGTGatgGTGAAAGTGCTGCCGCAGGTGGTGCCGatgTGCCTGCGTGTGCTGGTGAACATTGCGCGTCAGGGCGGCGCGAGCAGCCTGGAAAACACCGTGGATCTGCATATTAGCAACAGCCAGCCGCTGAGCCTGACCAGCGATCAGTATatgGCGTATCTGCAGGATCTGGCGGAAGATatgGATTTT

>IRCH-B4-5209

atgGGCACCCAGAAACCGCGTATTCTGCCGTGGCTGATTAGCCAGCTGGATCGTGGCGAACTGGAAGGCGTGGCGTGGCTGGGCGAAAGCCGTACCCGTTTTCGTATTCCGTGGAAACATGGCCTGCGTCAGGATGCGCAGCAGGAAGATTTTGGCATTTTTCAGGTGCGTGCGAGCCGTGAAGGCGCGTGGGCGGAAGCGAGCGGCGCGTATACCCCGGGCAAAGATAAACCGGATCTGCCGACCTGGAAACGTAACTTTCGTAGCGCGCTGAACCGTAAAGAAGTGCTGCGTCTGGCGGAAGATCATAGCAAAGATAGCCAGGATCCGCATAAAATTTATGAATTTGTGAACAGCGGCGTGCTGGAAGTGGGCGTGCGTGATATTCCGGAAACCGATACCGCGCAGGATAACGGCCGTCATAGCACCAGCGATACCCAGGTGCGTCGTCTGCCGAGCCATGCGGCGTTTAGCGTGCAGGAAGATATTGTGCAGAAACTGCTGAGCGATatgGATCTGAGCCCGGAAGGCGGCCCGAGCAACCTGACCatgACCAGCGAAAACCCGCCGCAGCTGCTGCTGAGCCCGGAAAGCGATATTCCGGCGCTGTGCCCGAACAGCGGCCTGAGCGAAAACCCGCTGAAACAGCTGCTGGCGAACGAAGAAAGCGATTGGGAATTTGAAGTGACCGCGTTTTATCGTGGCTGCCAGGTGTTTCAGCAGACCGTGTTTTGCCCGGGCGGCCTGCGTCTGGTGGGCAGCGAAGCGGGCGATCGTatgCTGCCGGGCCAGCCGATTCGTCTGCCGGATCCGGCGGCGAGCCTGGCGGATAAAAGCGTGACCGATTATGTGCAGTGCGTGCTGAGCTGCCTGGGCGGCGGCCTGGCGCTGTGGCGTGCGGGC---------------------CTGGGCCATTGCCATGTGTATTGGGCGATTGGCGAAGAACTGCTGCCGAGCTGCGGCCATAAACCGGATGGCGAAGTGCCGAAAGATCGTGAAGGCGGCGTGTTTAACCTGGGCCCGTTTATTACCCCGTGGCCGCCGGATCTGATTAACTTTACCGAAGGCAGCCGTCGTAGCCCGCTGTATACCCTGTGGTTTTGCGTGGGCCAGAGCTGGCCGCAGGATCAGCCGTGGATTAAACGTCTGGTGatgGTGAAAGTGCTGCCGCAGGTGGTGCCGatgTGCCTGCGTGTGCTGGTGAACATTGCGCGTCAGGGCGGCGCGAGCAGCCTGGAAAACACCGTGGATCTGCATATTAGCAACAGCCAGCCGCTGAGCCTGACCAGCGATCAGTATatgGCGTATCTGCAGGATCTGGCGGAAGATatgGATTTT

>IRCH-B5-5032

atgGGCACCCAGAAACCGCGTATTCTGCCGTGGCTGATTAGCCAGCTGGATCGTGGCGAACTGGAAGGCGTGGCGTGGCTGGGCGAAAGCCGTACCCGTTTTCGTATTCCGTGGAAACATGGCCTGCGTCAGGATGCGCAGCAGGAAGATTTTGGCATTTTTCAGGTGCGTGCGAGCCGTGAAGGCGCGTGGGCGGAAGCGAGCGGCGCGTATACCCCGGGCAAAGATAAACCGGATCTGCCGACCTGGAAACGTAACTTTCGTAGCGCGCTGAACCGTAAAGAAGTGCTGCGTCTGGCGGAAGATCATAGCAAAGATAGCCAGGATCCGCATAAAATTTATGAATTTGTGAACAGCGGCGTGCTGGAAGTGGGCGTGCGTGATATTCCGGAAACCGATACCGCGCAGGATAACGGCCGTCATAGCACCAGCGATACCCAGGTGCGTCGTCTGCCGAGCCATGCGGCGTTTAGCGTGCAGGAAGATATTGTGCAGAAACTGCTGAGCGATatgGATCTGAGCCCGGAAGGCGGCCCGAGCAACCTGACCatgACCAGCGAAAACCCGCCGCAGCTGCTGCTGAGCCCGGAAAGCGATATTCCGGCGCTGTGCCCGAACAGCGGCCTGAGCGAAAACCCGCTGAAACAGCTGCTGGCGAACGAAGAAAGCGATTGGGAATTTGAAGTGACCGCGTTTTATCGTGGCTGCCAGGTGTTTCAGCAGACCGTGTTTTGCCCGGGCGGCCTGCGTCTGGTGGGCAGCGAAGCGGGCGATCGTatgCTGCCGGGCCAGCCGATTCGTCTGCCGGATCCGGCGGCGAGCCTGGCGGATAAAAGCGTGACCGATTATGTGCAGTGCGTGCTGAGCTGCCTGGGCGGCGGCCTGGCGCTGTGGCGTGCGGGC---------------------CTGGGCCATTGCCATGTGTATTGGGCGATTGGCGAAGAACTGCTGCCGAGCTGCGGCCATAAACCGGATGGCGAAGTGCCGAAAGATCGTGAAGGCGGCGTGTTTAACCTGGGCCCGTTTATTACCCCGTGGCCGCCGGATCTGATTAACTTTACCGAAGGCAGCCGTCGTAGCCCGCTGTATACCCTGTGGTTTTGCGTGGGCCAGAGCTGGCCGCAGGATCAGCCGTGGATTAAACGTCTGGTGatgGTGAAAGTGCTGCCGCAGGTGGTGCCGatgTGCCTGCGTGTGCTGGTGAACATTGCGCGTCAGGGCGGCGCGAGCAGCCTGGAAAACACCGTGGATCTGCATATTAGCAACAGCCAGCCGCTGAGCCTGACCAGCGATCAGTATatgGCGTATCTGCAGGATCTGGCGGAAGATatgGATTTT

>IRCH-C3-5039

atgGGCACCCAGAAACCGCGTATTCTGCCGTGGCTGATTAGCCAGCTGGATCGTGGCGAACTGGAAGGCGTGGCGTGGCTGGGCGAAAGCCGTACCCGTTTTCGTATTCCGTGGAAACATGGCCTGCGTCAGGATGCGCAGCAGGAAGATTTTGGCATTTTTCAGGTGCGTGCGAGCCGTGAAGGCGCGTGGGCGGAAGCGAGCGGCGCGTATACCCCGGGCAAAGATAAACCGGATCTGCCGACCTGGAAACGTAACTTTCGTAGCGCGCTGAACCGTAAAGAAGTGCTGCGTCTGGCGGAAGATCATAGCAAAGATAGCCAGGATCCGCATAAAATTTATGAATTTGTGAACAGCGGCGTGCTGGAAGTGGGCGTGCGTGATATTCCGGAAACCGATACCGCGCAGGATAACGGCCGTCATAGCACCAGCGATACCCAGGTGCGTCGTCTGCCGAGCCATGCGGCGTTTAGCGTGCAGGAAGATATTGTGCAGAAACTGCTGAGCGATatgGATCTGAGCCCGGAAGGCGGCCCGAGCAACCTGACCatgACCAGCGAAAACCCGCCGCAGCTGCTGCTGAGCCCGGAAAGCGATATTCCGGCGCTGTGCCCGAACAGCGGCCTGAGCGAAAACCCGCTGAAACAGCTGCTGGCGAACGAAGAAAGCGATTGGGAATTTGAAGTGACCGCGTTTTATCGTGGCTGCCAGGTGTTTCAGCAGACCGTGTTTTGCCCGGGCGGCCTGCGTCTGGTGGGCAGCGAAGCGGGCGATCGTatgCTGCCGGGCCAGCCGATTCGTCTGCCGGATCCGGCGGCGAGCCTGGCGGATAAAAGCGTGACCGATTATGTGCAGTGCGTGCTGAGCTGCCTGGGCGGCGGCCTGGCGCTGTGGCGTGCGGGC---------------------CTGGGCCATTGCCATGTGTATTGGGCGATTGGCGAAGAACTGCTGCCGAGCTGCGGCCATAAACCGGATGGCGAAGTGCCGAAAGATCGTGAAGGCGGCGTGTTTAACCTGGGCCCGTTTATTACCCCGTGGCCGCCGGATCTGATTAACTTTACCGAAGGCAGCCGTCGTAGCCCGCTGTATACCCTGTGGTTTTGCGTGGGCCAGAGCTGGCCGCAGGATCAGCCGTGGATTAAACGTCTGGTGatgGTGAAAGTGCTGCCGCAGGTGGTGCCGatgTGCCTGCGTGTGCTGGTGAACATTGCGCGTCAGGGCGGCGCGAGCAGCCTGGAAAACACCGTGGATCTGCATATTAGCAACAGCCAGCCGCTGAGCCTGACCAGCGATCAGTATatgGCGTATCTGCAGGATCTGGCGGAAGATatgGATTTT

>IRCH-C5-5206

atgGGCACCCAGAAACCGCGTATTCTGCCGTGGCTGATTAGCCAGCTGGATCGTGGCGAACTGGAAGGCGTGGCGTGGCTGGGCGAAAGCCGTACCCGTTTTCGTATTCCGTGGAAACATGGCCTGCGTCAGGATGCGCAGCAGGAAGATTTTGGCATTTTTCAGGTGCGTGCGAGCCGTGAAGGCGCGTGGGCGGAAGCGAGCGGCGCGTATACCCCGGGCAAAGATAAACCGGATCTGCCGACCTGGAAACGTAACTTTCGTAGCGCGCTGAACCGTAAAGAAGTGCTGCGTCTGGCGGAAGATCATAGCAAAGATAGCCAGGATCCGCATAAAATTTATGAATTTGTGAACAGCGGCGTGCTGGAAGTGGGCGTGCGTGATATTCCGGAAACCGATACCGCGCAGGATAACGGCCGTCATAGCACCAGCGATACCCAGGTGCGTCGTCTGCCGAGCCATGCGGCGTTTAGCGTGCAGGAAGATATTGTGCAGAAACTGCTGAGCGATatgGATCTGAGCCCGGAAGGCGGCCCGAGCAACCTGACCatgACCAGCGAAAACCCGCCGCAGCTGCTGCTGAGCCCGGAAAGCGATATTCCGGCGCTGTGCCCGAACAGCGGCCTGAGCGAAAACCCGCTGAAACAGCTGCTGGCGAACGAAGAAAGCGATTGGGAATTTGAAGTGACCGCGTTTTATCGTGGCTGCCAGGTGTTTCAGCAGACCGTGTTTTGCCCGGGCGGCCTGCGTCTGGTGGGCAGCGAAGCGGGCGATCGTatgCTGCCGGGCCAGCCGATTCGTCTGCCGGATCCGGCGGCGAGCCTGGCGGATAAAAGCGTGACCGATTATGTGCAGTGCGTGCTGAGCTGCCTGGGCGGCGGCCTGGCGCTGTGGCGTGCGGGC---------------------CTGGGCCATTGCCATGTGTATTGGGCGATTGGCGAAGAACTGCTGCCGAGCTGCGGCCATAAACCGGATGGCGAAGTGCCGAAAGATCGTGAAGGCGGCGTGTTTAACCTGGGCCCGTTTATTACCCCGTGGCCGCCGGATCTGATTAACTTTACCGAAGGCAGCCGTCGTAGCCCGCTGTATACCCTGTGGTTTTGCGTGGGCCAGAGCTGGCCGCAGGATCAGCCGTGGATTAAACGTCTGGTGatgGTGAAAGTGCTGCCGCAGGTGGTGCCGatgTGCCTGCGTGTGCTGGTGAACATTGCGCGTCAGGGCGGCGCGAGCAGCCTGGAAAACACCGTGGATCTGCATATTAGCAACAGCCAGCCGCTGAGCCTGACCAGCGATCAGTATatgGCGTATCTGCAGGATCTGGCGGAAGATatgGATTTT

>IRCH-C6-5204

atgGGCACCCAGAAACCGCGTATTCTGCCGTGGCTGATTAGCCAGCTGGATCGTGGCGAACTGGAAGGCGTGGCGTGGCTGGGCGAAAGCCGTACCCGTTTTCGTATTCCGTGGAAACATGGCCTGCGTCAGGATGCGCAGCAGGAAGATTTTGGCATTTTTCAGGTGCGTGCGAGCCGTGAAGGCGCGTGGGCGGAAGCGAGCGGCGCGTATACCCCGGGCAAAGATAAACCGGATCTGCCGACCTGGAAACGTAACTTTCGTAGCGCGCTGAACCGTAAAGAAGTGCTGCGTCTGGCGGAAGATCATAGCAAAGATAGCCAGGATCCGCATAAAATTTATGAATTTGTGAACAGCGGCGTGCTGGAAGTGGGCGTGCGTGATATTCCGGAAACCGATACCGCGCAGGATAACGGCCGTCATAGCACCAGCGATACCCAGGTGCGTCGTCTGCCGAGCCATGCGGCGTTTAGCGTGCAGGAAGATATTGTGCAGAAACTGCTGAGCGATatgGATCTGAGCCCGGAAGGCGGCCCGAGCAACCTGACCatgACCAGCGAAAACCCGCCGCAGCTGCTGCTGAGCCCGGAAAGCGATATTCCGGCGCTGTGCCCGAACAGCGGCCTGAGCGAAAACCCGCTGAAACAGCTGCTGGCGAACGAAGAAAGCGATTGGGAATTTGAAGTGACCGCGTTTTATCGTGGCTGCCAGGTGTTTCAGCAGACCGTGTTTTGCCCGGGCGGCCTGCGTCTGGTGGGCAGCGAAGCGGGCGATCGTatgCTGCCGGGCCAGCCGATTCGTCTGCCGGATCCGGCGGCGAGCCTGGCGGATAAAAGCGTGACCGATTATGTGCAGTGCGTGCTGAGCTGCCTGGGCGGCGGCCTGGCGCTGTGGCGTGCGGGC---------------------CTGGGCCATTGCCATGTGTATTGGGCGATTGGCGAAGAACTGCTGCCGAGCTGCGGCCATAAACCGGATGGCGAAGTGCCGAAAGATCGTGAAGGCGGCGTGTTTAACCTGGGCCCGTTTATTACCCCGTGGCCGCCGGATCTGATTAACTTTACCGAAGGCAGCCGTCGTAGCCCGCTGTATACCCTGTGGTTTTGCGTGGGCCAGAGCTGGCCGCAGGATCAGCCGTGGATTAAACGTCTGGTGatgGTGAAAGTGCTGCCGCAGGTGGTGCCGatgTGCCTGCGTGTGCTGGTGAACATTGCGCGTCAGGGCGGCGCGAGCAGCCTGGAAAACACCGTGGATCTGCATATTAGCAACAGCCAGCCGCTGAGCCTGACCAGCGATCAGTATatgGCGTATCTGCAGGATCTGGCGGAAGATatgGATTTT

>IRCH-C7-5144

atgGGCACCCAGAAACCGCGTATTCTGCCGTGGCTGATTAGCCAGCTGGATCGTGGCGAACTGGAAGGCGTGGCGTGGCTGGGCGAAAGCCGTACCCGTTTTCGTATTCCGTGGAAACATGGCCTGCGTCAGGATGCGCAGCAGGAAGATTTTGGCATTTTTCAGGTGCGTGCGAGCCGTGAAGGCGCGTGGGCGGAAGCGAGCGGCGCGTATACCCCGGGCAAAGATAAACCGGATCTGCCGACCTGGAAACGTAACTTTCGTAGCGCGCTGAACCGTAAAGAAGTGCTGCGTCTGGCGGAAGATCATAGCAAAGATAGCCAGGATCCGCATAAAATTTATGAATTTGTGAACAGCGGCGTGCTGGAAGTGGGCGTGCGTGATATTCCGGAAACCGATACCGCGCAGGATAACGGCCGTCATAGCACCAGCGATACCCAGGTGCGTCGTCTGCCGAGCCATGCGGCGTTTAGCGTGCAGGAAGATATTGTGCAGAAACTGCTGAGCGATatgGATCTGAGCCCGGAAGGCGGCCCGAGCAACCTGACCatgACCAGCGAAAACCCGCCGCAGCTGCTGCTGAGCCCGGAAAGCGATATTCCGGCGCTGTGCCCGAACAGCGGCCTGAGCGAAAACCCGCTGAAACAGCTGCTGGCGAACGAAGAAAGCGATTGGGAATTTGAAGTGACCGCGTTTTATCGTGGCTGCCAGGTGTTTCAGCAGACCGTGTTTTGCCCGGGCGGCCTGCGTCTGGTGGGCAGCGAAGCGGGCGATCGTatgCTGCCGGGCCAGCCGATTCGTCTGCCGGATCCGGCGGCGAGCCTGGCGGATAAAAGCGTGACCGATTATGTGCAGTGCGTGCTGAGCTGCCTGGGCGGCGGCCTGGCGCTGTGGCGTGCGGGC---------------------CTGGGCCATTGCCATGTGTATTGGGCGATTGGCGAAGAACTGCTGCCGAGCTGCGGCCATAAACCGGATGGCGAAGTGCCGAAAGATCGTGAAGGCGGCGTGTTTAACCTGGGCCCGTTTATTACCCCGTGGCCGCCGGATCTGATTAACTTTACCGAAGGCAGCCGTCGTAGCCCGCTGTATACCCTGTGGTTTTGCGTGGGCCAGAGCTGGCCGCAGGATCAGCCGTGGATTAAACGTCTGGTGatgGTGAAAGTGCTGCCGCAGGTGGTGCCGatgTGCCTGCGTGTGCTGGTGAACATTGCGCGTCAGGGCGGCGCGAGCAGCCTGGAAAACACCGTGGATCTGCATATTAGCAACAGCCAGCCGCTGAGCCTGACCAGCGATCAGTATatgGCGTATCTGCAGGATCTGGCGGAAGATatgGATTTT

>IRCH-D5-5240

atgGGCACCCAGAAACCGCGTATTCTGCCGTGGCTGATTAGCCAGCTGGATCGTGGCGAACTGGAAGGCGTGGCGTGGCTGGGCGAAAGCCGTACCCGTTTTCGTATTCCGTGGAAACATGGCCTGCGTCAGGATGCGCAGCAGGAAGATTTTGGCATTTTTCAGGTGCGTGCGAGCCGTGAAGGCGCGTGGGCGGAAGCGAGCGGCGCGTATACCCCGGGCAAAGATAAACCGGATCTGCCGACCTGGAAACGTAACTTTCGTAGCGCGCTGAACCGTAAAGAAGTGCTGCGTCTGGCGGAAGATCATAGCAAAGATAGCCAGGATCCGCATAAAATTTATGAATTTGTGAACAGCGGCGTGCTGGAAGTGGGCGTGCGTGATATTCCGGAAACCGATACCGCGCAGGATAACGGCCGTCATAGCACCAGCGATACCCAGGTGCGTCGTCTGCCGAGCCATGCGGCGTTTAGCGTGCAGGAAGATATTGTGCAGAAACTGCTGAGCGATatgGATCTGAGCCCGGAAGGCGGCCCGAGCAACCTGACCatgACCAGCGAAAACCCGCCGCAGCTGCTGCTGAGCCCGGAAAGCGATATTCCGGCGCTGTGCCCGAACAGCGGCCTGAGCGAAAACCCGCTGAAACAGCTGCTGGCGAACGAAGAAAGCGATTGGGAATTTGAAGTGACCGCGTTTTATCGTGGCTGCCAGGTGTTTCAGCAGACCGTGTTTTGCCCGGGCGGCCTGCGTCTGGTGGGCAGCGAAGCGGGCGATCGTatgCTGCCGGGCCAGCCGATTCGTCTGCCGGATCCGGCGGCGAGCCTGGCGGATAAAAGCGTGACCGATTATGTGCAGTGCGTGCTGAGCTGCCTGGGCGGCGGCCTGGCGCTGTGGCGTGCGGGC---------------------CTGGGCCATTGCCATGTGTATTGGGCGATTGGCGAAGAACTGCTGCCGAGCTGCGGCCATAAACCGGATGGCGAAGTGCCGAAAGATCGTGAAGGCGGCGTGTTTAACCTGGGCCCGTTTATTACCCCGTGGCCGCCGGATCTGATTAACTTTACCGAAGGCAGCCGTCGTAGCCCGCTGTATACCCTGTGGTTTTGCGTGGGCCAGAGCTGGCCGCAGGATCAGCCGTGGATTAAACGTCTGGTGatgGTGAAAGTGCTGCCGCAGGTGGTGCCGatgTGCCTGCGTGTGCTGGTGAACATTGCGCGTCAGGGCGGCGCGAGCAGCCTGGAAAACACCGTGGATCTGCATATTAGCAACAGCCAGCCGCTGAGCCTGACCAGCGATCAGTATatgGCGTATCTGCAGGATCTGGCGGAAGATatgGATTTT

>IRCH-D6-5189

atgGGCACCCAGAAACCGCGTATTCTGCCGTGGCTGATTAGCCAGCTGGATCGTGGCGAACTGGAAGGCGTGGCGTGGCTGGGCGAAAGCCGTACCCGTTTTCGTATTCCGTGGAAACATGGCCTGCGTCAGGATGCGCAGCAGGAAGATTTTGGCATTTTTCAGGTGCGTGCGAGCCGTGAAGGCGCGTGGGCGGAAGCGAGCGGCGCGTATACCCCGGGCAAAGATAAACCGGATCTGCCGACCTGGAAACGTAACTTTCGTAGCGCGCTGAACCGTAAAGAAGTGCTGCGTCTGGCGGAAGATCATAGCAAAGATAGCCAGGATCCGCATAAAATTTATGAATTTGTGAACAGCGGCGTGCTGGAAGTGGGCGTGCGTGATATTCCGGAAACCGATACCGCGCAGGATAACGGCCGTCATAGCACCAGCGATACCCAGGTGCGTCGTCTGCCGAGCCATGCGGCGTTTAGCGTGCAGGAAGATATTGTGCAGAAACTGCTGAGCGATatgGATCTGAGCCCGGAAGGCGGCCCGAGCAACCTGACCatgACCAGCGAAAACCCGCCGCAGCTGCTGCTGAGCCCGGAAAGCGATATTCCGGCGCTGTGCCCGAACAGCGGCCTGAGCGAAAACCCGCTGAAACAGCTGCTGGCGAACGAAGAAAGCGATTGGGAATTTGAAGTGACCGCGTTTTATCGTGGCTGCCAGGTGTTTCAGCAGACCGTGTTTTGCCCGGGCGGCCTGCGTCTGGTGGGCAGCGAAGCGGGCGATCGTatgCTGCCGGGCCAGCCGATTCGTCTGCCGGATCCGGCGGCGAGCCTGGCGGATAAAAGCGTGACCGATTATGTGCAGTGCGTGCTGAGCTGCCTGGGCGGCGGCCTGGCGCTGTGGCGTGCGGGC---------------------CTGGGCCATTGCCATGTGTATTGGGCGATTGGCGAAGAACTGCTGCCGAGCTGCGGCCATAAACCGGATGGCGAAGTGCCGAAAGATCGTGAAGGCGGCGTGTTTAACCTGGGCCCGTTTATTACCCCGTGGCCGCCGGATCTGATTAACTTTACCGAAGGCAGCCGTCGTAGCCCGCTGTATACCCTGTGGTTTTGCGTGGGCCAGAGCTGGCCGCAGGATCAGCCGTGGATTAAACGTCTGGTGatgGTGAAAGTGCTGCCGCAGGTGGTGCCGatgTGCCTGCGTGTGCTGGTGAACATTGCGCGTCAGGGCGGCGCGAGCAGCCTGGAAAACACCGTGGATCTGCATATTAGCAACAGCCAGCCGCTGAGCCTGACCAGCGATCAGTATatgGCGTATCTGCAGGATCTGGCGGAAGATatgGATTTT

>IRCH-D7-5132

atgGGCACCCAGAAACCGCGTATTCTGCCGTGGCTGATTAGCCAGCTGGATCGTGGCGAACTGGAAGGCGTGGCGTGGCTGGGCGAAAGCCGTACCCGTTTTCGTATTCCGTGGAAACATGGCCTGCGTCAGGATGCGCAGCAGGAAGATTTTGGCATTTTTCAGGTGCGTGCGAGCCGTGAAGGCGCGTGGGCGGAAGCGAGCGGCGCGTATACCCCGGGCAAAGATAAACCGGATCTGCCGACCTGGAAACGTAACTTTCGTAGCGCGCTGAACCGTAAAGAAGTGCTGCGTCTGGCGGAAGATCATAGCAAAGATAGCCAGGATCCGCATAAAATTTATGAATTTGTGAACAGCGGCGTGCTGGAAGTGGGCGTGCGTGATATTCCGGAAACCGATACCGCGCAGGATAACGGCCGTCATAGCACCAGCGATACCCAGGTGCGTCGTCTGCCGAGCCATGCGGCGTTTAGCGTGCAGGAAGATATTGTGCAGAAACTGCTGAGCGATatgGATCTGAGCCCGGAAGGCGGCCCGAGCAACCTGACCatgACCAGCGAAAACCCGCCGCAGCTGCTGCTGAGCCCGGAAAGCGATATTCCGGCGCTGTGCCCGAACAGCGGCCTGAGCGAAAACCCGCTGAAACAGCTGCTGGCGAACGAAGAAAGCGATTGGGAATTTGAAGTGACCGCGTTTTATCGTGGCTGCCAGGTGTTTCAGCAGACCGTGTTTTGCCCGGGCGGCCTGCGTCTGGTGGGCAGCGAAGCGGGCGATCGTatgCTGCCGGGCCAGCCGATTCGTCTGCCGGATCCGGCGGCGAGCCTGGCGGATAAAAGCGTGACCGATTATGTGCAGTGCGTGCTGAGCTGCCTGGGCGGCGGCCTGGCGCTGTGGCGTGCGGGC---------------------CTGGGCCATTGCCATGTGTATTGGGCGATTGGCGAAGAACTGCTGCCGAGCTGCGGCCATAAACCGGATGGCGAAGTGCCGAAAGATCGTGAAGGCGGCGTGTTTAACCTGGGCCCGTTTATTACCCCGTGGCCGCCGGATCTGATTAACTTTACCGAAGGCAGCCGTCGTAGCCCGCTGTATACCCTGTGGTTTTGCGTGGGCCAGAGCTGGCCGCAGGATCAGCCGTGGATTAAACGTCTGGTGatgGTGAAAGTGCTGCCGCAGGTGGTGCCGatgTGCCTGCGTGTGCTGGTGAACATTGCGCGTCAGGGCGGCGCGAGCAGCCTGGAAAACACCGTGGATCTGCATATTAGCAACAGCCAGCCGCTGAGCCTGACCAGCGATCAGTATatgGCGTATCTGCAGGATCTGGCGGAAGATatgGATTTT

>IRCH-E5-5053

atgGGCACCCAGAAACCGCGTATTCTGCCGTGGCTGATTAGCCAGCTGGATCGTGGCGAACTGGAAGGCGTGGCGTGGCTGGGCGAAAGCCGTACCCGTTTTCGTATTCCGTGGAAACATGGCCTGCGTCAGGATGCGCAGCAGGAAGATTTTGGCATTTTTCAGGTGCGTGCGAGCCGTGAAGGCGCGTGGGCGGAAGCGAGCGGCGCGTATACCCCGGGCAAAGATAAACCGGATCTGCCGACCTGGAAACGTAACTTTCGTAGCGCGCTGAACCGTAAAGAAGTGCTGCGTCTGGCGGAAGATCATAGCAAAGATAGCCAGGATCCGCATAAAATTTATGAATTTGTGAACAGCGGCGTGCTGGAAGTGGGCGTGCGTGATATTCCGGAAACCGATACCGCGCAGGATAACGGCCGTCATAGCACCAGCGATACCCAGGTGCGTCGTCTGCCGAGCCATGCGGCGTTTAGCGTGCAGGAAGATATTGTGCAGAAACTGCTGAGCGATatgGATCTGAGCCCGGAAGGCGGCCCGAGCAACCTGACCatgACCAGCGAAAACCCGCCGCAGCTGCTGCTGAGCCCGGAAAGCGATATTCCGGCGCTGTGCCCGAACAGCGGCCTGAGCGAAAACCCGCTGAAACAGCTGCTGGCGAACGAAGAAAGCGATTGGGAATTTGAAGTGACCGCGTTTTATCGTGGCTGCCAGGTGTTTCAGCAGACCGTGTTTTGCCCGGGCGGCCTGCGTCTGGTGGGCAGCGAAGCGGGCGATCGTatgCTGCCGGGCCAGCCGATTCGTCTGCCGGATCCGGCGGCGAGCCTGGCGGATAAAAGCGTGACCGATTATGTGCAGTGCGTGCTGAGCTGCCTGGGCGGCGGCCTGGCGCTGTGGCGTGCGGGC---------------------CTGGGCCATTGCCATGTGTATTGGGCGATTGGCGAAGAACTGCTGCCGAGCTGCGGCCATAAACCGGATGGCGAAGTGCCGAAAGATCGTGAAGGCGGCGTGTTTAACCTGGGCCCGTTTATTACCCCGTGGCCGCCGGATCTGATTAACTTTACCGAAGGCAGCCGTCGTAGCCCGCTGTATACCCTGTGGTTTTGCGTGGGCCAGAGCTGGCCGCAGGATCAGCCGTGGATTAAACGTCTGGTGatgGTGAAAGTGCTGCCGCAGGTGGTGCCGatgTGCCTGCGTGTGCTGGTGAACATTGCGCGTCAGGGCGGCGCGAGCAGCCTGGAAAACACCGTGGATCTGCATATTAGCAACAGCCAGCCGCTGAGCCTGACCAGCGATCAGTATatgGCGTATCTGCAGGATCTGGCGGAAGATatgGATTTT

>IRCH-E6-5087

atgGGCACCCAGAAACCGCGTATTCTGCCGTGGCTGATTAGCCAGCTGGATCGTGGCGAACTGGAAGGCGTGGCGTGGCTGGGCGAAAGCCGTACCCGTTTTCGTATTCCGTGGAAACATGGCCTGCGTCAGGATGCGCAGCAGGAAGATTTTGGCATTTTTCAGGTGCGTGCGAGCCGTGAAGGCGCGTGGGCGGAAGCGAGCGGCGCGTATACCCCGGGCAAAGATAAACCGGATCTGCCGACCTGGAAACGTAACTTTCGTAGCGCGCTGAACCGTAAAGAAGTGCTGCGTCTGGCGGAAGATCATAGCAAAGATAGCCAGGATCCGCATAAAATTTATGAATTTGTGAACAGCGGCGTGCTGGAAGTGGGCGTGCGTGATATTCCGGAAACCGATACCGCGCAGGATAACGGCCGTCATAGCACCAGCGATACCCAGGTGCGTCGTCTGCCGAGCCATGCGGCGTTTAGCGTGCAGGAAGATATTGTGCAGAAACTGCTGAGCGATatgGATCTGAGCCCGGAAGGCGGCCCGAGCAACCTGACCatgACCAGCGAAAACCCGCCGCAGCTGCTGCTGAGCCCGGAAAGCGATATTCCGGCGCTGTGCCCGAACAGCGGCCTGAGCGAAAACCCGCTGAAACAGCTGCTGGCGAACGAAGAAAGCGATTGGGAATTTGAAGTGACCGCGTTTTATCGTGGCTGCCAGGTGTTTCAGCAGACCGTGTTTTGCCCGGGCGGCCTGCGTCTGGTGGGCAGCGAAGCGGGCGATCGTatgCTGCCGGGCCAGCCGATTCGTCTGCCGGATCCGGCGGCGAGCCTGGCGGATAAAAGCGTGACCGATTATGTGCAGTGCGTGCTGAGCTGCCTGGGCGGCGGCCTGGCGCTGTGGCGTGCGGGC---------------------CTGGGCCATTGCCATGTGTATTGGGCGATTGGCGAAGAACTGCTGCCGAGCTGCGGCCATAAACCGGATGGCGAAGTGCCGAAAGATCGTGAAGGCGGCGTGTTTAACCTGGGCCCGTTTATTACCCCGTGGCCGCCGGATCTGATTAACTTTACCGAAGGCAGCCGTCGTAGCCCGCTGTATACCCTGTGGTTTTGCGTGGGCCAGAGCTGGCCGCAGGATCAGCCGTGGATTAAACGTCTGGTGatgGTGAAAGTGCTGCCGCAGGTGGTGCCGatgTGCCTGCGTGTGCTGGTGAACATTGCGCGTCAGGGCGGCGCGAGCAGCCTGGAAAACACCGTGGATCTGCATATTAGCAACAGCCAGCCGCTGAGCCTGACCAGCGATCAGTATatgGCGTATCTGCAGGATCTGGCGGAAGATatgGATTTT

>IRCH-E7-5193

atgGGCACCCAGAAACCGCGTATTCTGCCGTGGCTGATTAGCCAGCTGGATCGTGGCGAACTGGAAGGCGTGGCGTGGCTGGGCGAAAGCCGTACCCGTTTTCGTATTCCGTGGAAACATGGCCTGCGTCAGGATGCGCAGCAGGAAGATTTTGGCATTTTTCAGGTGCGTGCGAGCCGTGAAGGCGCGTGGGCGGAAGCGAGCGGCGCGTATACCCCGGGCAAAGATAAACCGGATCTGCCGACCTGGAAACGTAACTTTCGTAGCGCGCTGAACCGTAAAGAAGTGCTGCGTCTGGCGGAAGATCATAGCAAAGATAGCCAGGATCCGCATAAAATTTATGAATTTGTGAACAGCGGCGTGCTGGAAGTGGGCGTGCGTGATATTCCGGAAACCGATACCGCGCAGGATAACGGCCGTCATAGCACCAGCGATACCCAGGTGCGTCGTCTGCCGAGCCATGCGGCGTTTAGCGTGCAGGAAGATATTGTGCAGAAACTGCTGAGCGATatgGATCTGAGCCCGGAAGGCGGCCCGAGCAACCTGACCatgACCAGCGAAAACCCGCCGCAGCTGCTGCTGAGCCCGGAAAGCGATATTCCGGCGCTGTGCCCGAACAGCGGCCTGAGCGAAAACCCGCTGAAACAGCTGCTGGCGAACGAAGAAAGCGATTGGGAATTTGAAGTGACCGCGTTTTATCGTGGCTGCCAGGTGTTTCAGCAGACCGTGTTTTGCCCGGGCGGCCTGCGTCTGGTGGGCAGCGAAGCGGGCGATCGTatgCTGCCGGGCCAGCCGATTCGTCTGCCGGATCCGGCGGCGAGCCTGGCGGATAAAAGCGTGACCGATTATGTGCAGTGCGTGCTGAGCTGCCTGGGCGGCGGCCTGGCGCTGTGGCGTGCGGGC---------------------CTGGGCCATTGCCATGTGTATTGGGCGATTGGCGAAGAACTGCTGCCGAGCTGCGGCCATAAACCGGATGGCGAAGTGCCGAAAGATCGTGAAGGCGGCGTGTTTAACCTGGGCCCGTTTATTACCCCGTGGCCGCCGGATCTGATTAACTTTACCGAAGGCAGCCGTCGTAGCCCGCTGTATACCCTGTGGTTTTGCGTGGGCCAGAGCTGGCCGCAGGATCAGCCGTGGATTAAACGTCTGGTGatgGTGAAAGTGCTGCCGCAGGTGGTGCCGatgTGCCTGCGTGTGCTGGTGAACATTGCGCGTCAGGGCGGCGCGAGCAGCCTGGAAAACACCGTGGATCTGCATATTAGCAACAGCCAGCCGCTGAGCCTGACCAGCGATCAGTATatgGCGTATCTGCAGGATCTGGCGGAAGATatgGATTTT

>IRCH-F11-5140

atgGGCACCCAGAAACCGCGTATTCTGCCGTGGCTGATTAGCCAGCTGGATCGTGGCGAACTGGAAGGCGTGGCGTGGCTGGGCGAAAGCCGTACCCGTTTTCGTATTCCGTGGAAACATGGCCTGCGTCAGGATGCGCAGCAGGAAGATTTTGGCATTTTTCAGGTGCGTGCGAGCCGTGAAGGCGCGTGGGCGGAAGCGAGCGGCGCGTATACCCCGGGCAAAGATAAACCGGATCTGCCGACCTGGAAACGTAACTTTCGTAGCGCGCTGAACCGTAAAGAAGTGCTGCGTCTGGCGGAAGATCATAGCAAAGATAGCCAGGATCCGCATAAAATTTATGAATTTGTGAACAGCGGCGTGCTGGAAGTGGGCGTGCGTGATATTCCGGAAACCGATACCGCGCAGGATAACGGCCGTCATAGCACCAGCGATACCCAGGTGCGTCGTCTGCCGAGCCATGCGGCGTTTAGCGTGCAGGAAGATATTGTGCAGAAACTGCTGAGCGATatgGATCTGAGCCCGGAAGGCGGCCCGAGCAACCTGACCatgACCAGCGAAAACCCGCCGCAGCTGCTGCTGAGCCCGGAAAGCGATATTCCGGCGCTGTGCCCGAACAGCGGCCTGAGCGAAAACCCGCTGAAACAGCTGCTGGCGAACGAAGAAAGCGATTGGGAATTTGAAGTGACCGCGTTTTATCGTGGCTGCCAGGTGTTTCAGCAGACCGTGTTTTGCCCGGGCGGCCTGCGTCTGGTGGGCAGCGAAGCGGGCGATCGTatgCTGCCGGGCCAGCCGATTCGTCTGCCGGATCCGGCGGCGAGCCTGGCGGATAAAAGCGTGACCGATTATGTGCAGTGCGTGCTGAGCTGCCTGGGCGGCGGCCTGGCGCTGTGGCGTGCGGGC---------------------CTGGGCCATTGCCATGTGTATTGGGCGATTGGCGAAGAACTGCTGCCGAGCTGCGGCCATAAACCGGATGGCGAAGTGCCGAAAGATCGTGAAGGCGGCGTGTTTAACCTGGGCCCGTTTATTACCCCGTGGCCGCCGGATCTGATTAACTTTACCGAAGGCAGCCGTCGTAGCCCGCTGTATACCCTGTGGTTTTGCGTGGGCCAGAGCTGGCCGCAGGATCAGCCGTGGATTAAACGTCTGGTGatgGTGAAAGTGCTGCCGCAGGTGGTGCCGatgTGCCTGCGTGTGCTGGTGAACATTGCGCGTCAGGGCGGCGCGAGCAGCCTGGAAAACACCGTGGATCTGCATATTAGCAACAGCCAGCCGCTGAGCCTGACCAGCGATCAGTATatgGCGTATCTGCAGGATCTGGCGGAAGATatgGATTTT

>IRCH-F3-5044

atgGGCACCCAGAAACCGCGTATTCTGCCGTGGCTGATTAGCCAGCTGGATCGTGGCGAACTGGAAGGCGTGGCGTGGCTGGGCGAAAGCCGTACCCGTTTTCGTATTCCGTGGAAACATGGCCTGCGTCAGGATGCGCAGCAGGAAGATTTTGGCATTTTTCAGGTGCGTGCGAGCCGTGAAGGCGCGTGGGCGGAAGCGAGCGGCGCGTATACCCCGGGCAAAGATAAACCGGATCTGCCGACCTGGAAACGTAACTTTCGTAGCGCGCTGAACCGTAAAGAAGTGCTGCGTCTGGCGGAAGATCATAGCAAAGATAGCCAGGATCCGCATAAAATTTATGAATTTGTGAACAGCGGCGTGCTGGAAGTGGGCGTGCGTGATATTCCGGAAACCGATACCGCGCAGGATAACGGCCGTCATAGCACCAGCGATACCCAGGTGCGTCGTCTGCCGAGCCATGCGGCGTTTAGCGTGCAGGAAGATATTGTGCAGAAACTGCTGAGCGATatgGATCTGAGCCCGGAAGGCGGCCCGAGCAACCTGACCatgACCAGCGAAAACCCGCCGCAGCTGCTGCTGAGCCCGGAAAGCGATATTCCGGCGCTGTGCCCGAACAGCGGCCTGAGCGAAAACCCGCTGAAACAGCTGCTGGCGAACGAAGAAAGCGATTGGGAATTTGAAGTGACCGCGTTTTATCGTGGCTGCCAGGTGTTTCAGCAGACCGTGTTTTGCCCGGGCGGCCTGCGTCTGGTGGGCAGCGAAGCGGGCGATCGTatgCTGCCGGGCCAGCCGATTCGTCTGCCGGATCCGGCGGCGAGCCTGGCGGATAAAAGCGTGACCGATTATGTGCAGTGCGTGCTGAGCTGCCTGGGCGGCGGCCTGGCGCTGTGGCGTGCGGGC---------------------CTGGGCCATTGCCATGTGTATTGGGCGATTGGCGAAGAACTGCTGCCGAGCTGCGGCCATAAACCGGATGGCGAAGTGCCGAAAGATCGTGAAGGCGGCGTGTTTAACCTGGGCCCGTTTATTACCCCGTGGCCGCCGGATCTGATTAACTTTACCGAAGGCAGCCGTCGTAGCCCGCTGTATACCCTGTGGTTTTGCGTGGGCCAGAGCTGGCCGCAGGATCAGCCGTGGATTAAACGTCTGGTGatgGTGAAAGTGCTGCCGCAGGTGGTGCCGatgTGCCTGCGTGTGCTGGTGAACATTGCGCGTCAGGGCGGCGCGAGCAGCCTGGAAAACACCGTGGATCTGCATATTAGCAACAGCCAGCCGCTGAGCCTGACCAGCGATCAGTATatgGCGTATCTGCAGGATCTGGCGGAAGATatgGATTTT

>Capra_aegagrus

atgGGCACCCAGAAACCGCGTATTCTGCCGTGGCTGATTAGCCAGCTGGATCGTGGCGAACTGGAAGGCGTGGCGTGGCTGGGCGAAAGCCGTACCCGTTTTCGTATTCCGTGGAAACATGGCCTGCGTCAGGATGCGCAGCAGGAAGATTTTGGCATTTTTCAGGTGCGTGCGAGCCGTGAAGGCGCGTGGGCGGAAGCGAGCGGCGCGTATACCCCGGGCAAAGATAAACCGGATCTGCCGACCTGGAAACGTAACTTTCGTAGCGCGCTGAACCGTAAAGAAGTGCTGCGTCTGGCGGAAGATCATAGCAAAGATAGCCAGGATCCGCATAAAATTTATGAATTTGTGAACAGCGGCGTGCTGGAAGTG------------------------------------------------------------------------------------------------------GTGCAGGAAGATATTGTGCAGAAACTGCTGAGCGATatgGATCTGAGCCCGGAAGGCGGCCCGAGCAACCTGACCatgACCAGCGAAAACCCGCCGCAGCTGCTGCTGAGCCCGGAAAGCGATATTCCGGCGCTGTGCCCGAACTGGGGCCTGAGCGAAAACCCGCTGAAACAGCTGCTGGCGAACGAAGAAAGCGATTGGGAATTTGAAGTGACCGCGTTTTATCGTGGCTGCCAGGTGTTTCAGCAGACCGTGTTTTGCCCGGGCGGCCTGCGTCTGGTGGGCAGCGAAGCGGGCGATCGTatgCTGCCGGGCCAGCCGATTCGTCTGCCGGATCCGGCGGCGAGCCTGGCGGATAAAAGCGTGACCGATTATGTGCAGTGCGTGCTGAGCTGCCTGGGCGGCGGCCTGGCGCTGTGGCGTGCGGGCCAGTGGCTGTGCGCGCAGCGTCTGGGCCATTGCCATGTGTATTGGGCGATTGGCGAAGAACTGCTGCCGAGCTGCGGCCATAAACCGGATGGCGAAGTGCCGAAAGATCGTGAAGGCGGCGTGTTTAACCTGGGCCCGTTTATTACCCCGTGGCCGCCGGATCTGATTACCTTTACCGAAGGCAGCCGTCGTAGCCCGCTGTATACCCTGTGGTTTTGCGTGGGCCAGAGCTGGCCGCAGGATCAGCCGTGGATTAAACGTCTGGTGatgGTGAAAGTGCTGCCGCAGGTGGTGCCGatgTGCCTGCGTGTGCTGGTGGATATTGCGCGTCAGGGCGGCGCGAGCAGCCTGGAAAACACCGTGGATCTGCATATTAGCAACAGCCAGCCGCTGAGCCTGACCAGCGATCAGTATatgGCGTATCTGCAGGATCTGGCGGAAGATatgGATTTT

>Ethiopian_Borena

atgGGCACCCAGAAACCGCGTATTCTGCCGTGGCTGATTAGCCAGCTGGATCGTGGCGAACTGGAAGGCGTGGCGTGGCTGGGCGAAAGCCGTACCCGTTTTCGTATTCCGTGGAAACATGGCCTGCGTCAGGATGCGCAGCAGGAAGATTTTGGCATTTTTCAGGTGCGTGCGAGCCGTGAAGGCGCGTGGGCGGAAGCGAGCGGCGCGTATACCCCGGGCAAAGATAAACCGGATCTGCCGACCTGGAAACGTAACTTTCGTAGCGCGCTGAACCGTAAAGAAGTGCTGCGTCTGGCGGAAGATCATAGCAAAGATAGCCAGGATCCGCATAAAATTTATGAATTTGTGAACAGCGGCGTGCTGGAAGTGGGCGTGCGTGATATTCCGGAAACCGATACCGCGCAGGATAACGGCCGTCATAGCACCAGCGATACCCAGGTGCGTCGTCTGCCGAGCCATGCGGCGTTTAGCGTGCAGGAAGATATTGTGCAGAAACTGCTGAGCGATatgGATCTGAGCCCGGAAGGCGGCCCGAGCAACCTGACCatgACCAGCGAAAACCCGCCGCAGCTGCTGCTGAGCCCGGAAAGCGATATTCCGGCGCTGTGCCCGAACAGCGGCCTGAGCGAAAACCCGCTGAAACAGCTGCTGGCGAACGAAGAAAGCGATTGGGAATTTGAAGTGACCGCGTTTTATCGTGGCTGCCAGGTGTTTCAGCAGACCGTGTTTTGCCCGGGCGGCCTGCGTCTGGTGGGCAGCGAAGCGGGCGATCGTatgCTGCCGGGCCAGCCGATTCGTCTGCCGGATCCGGCGGCGAGCCTGGCGGATAAAAGCGTGACCGATTATGTGCAGTGCGTGCTGAGCTGCCTGGGCGGCGGCCTGGCGCTGTGGCGTGCGGGCCAGTGGCTGTGCGCGCAGCGTCTGGGCCATTGCCATGTGTATTGGGCGATTGGCGAAGAACTGCTGCCGAGCTGCGGCCATAAACCGGATGGCGAAGTGCCGAAAGATCGTGAAGGCGGCGTGTTTAACCTGGGCCCGTTTATTACCCCGTGGCCGCCGGATCTGATTAACTTTACCGAAGGCAGCCGTCGTAGCCCGCTGTATACCCTGTGGTTTTGCGTGGGCCAGAGCTGGCCGCAGGATCAGCCGTGGATTAAACGTCTGGTGatgGTGAAAGTGCTGCCGCAGGTGGTGCCGatgTGCCTGCGTGTGCTGGTGAACATTGCGCGTCAGGGCGGCGCGAGCAGCCTGGAAAACACCGTGGATCTGCATATTAGCAACAGCCAGCCGCTGAGCCTGACCAGCGATCAGTATatgGCGTATCTGCAGGATCTGGCGGAAGATatgGATTTT

>Ethiopian_Somali

atgGGCACCCAGAAACCGCGTATTCTGCCGTGGCTGATTAGCCAGCTGGATCGTGGCGAACTGGAAGGCGTGGCGTGGCTGGGCGAAAGCCGTACCCGTTTTCGTATTCCGTGGAAACATGGCCTGCGTCAGGATGCGCAGCAGGAAGATTTTGGCATTTTTCAGGTGCGTGCGAGCCGTGAAGGCGCGTGGGCGGAAGCGAGCGGCGCGTATACCCCGGGCAAAGATAAACCGGATCTGCCGACCTGGAAACGTAACTTTCGTAGCGCGCTGAACCGTAAAGAAGTGCTGCGTCTGGCGGAAGATCATAGCAAAGATAGCCAGGATCCGCATAAAATTTATGAATTTGTGAACAGCGGCGTGCTGGAAGTGGGCGTGCGTGATATTCCGGAAACCGATACCGCGCAGGATAACGGCCGTCATAGCACCAGCGATACCCAGGTGCGTCGTCTGCCGAGCCATGCGGCGTTTAGCGTGCAGGAAGATATTGTGCAGAAACTGCTGAGCGATatgGATCTGAGCCCGGAAGGCGGCCCGAGCAACCTGACCatgACCAGCGAAAACCCGCCGCAGCTGCTGCTGAGCCCGGAAAGCGATATTCCGGCGCTGTGCCCGAACAGCGGCCTGAGCGAAAACCCGCTGAAACAGCTGCTGGCGAACGAAGAAAGCGATTGGGAATTTGAAGTGACCGCGTTTTATCGTGGCTGCCAGGTGTTTCAGCAGACCGTGTTTTGCCCGGGCGGCCTGCGTCTGGTGGGCAGCGAAGCGGGCGATCGTatgCTGCCGGGCCAGCCGATTCGTCTGCCGGATCCGGCGGCGAGCCTGGCGGATAAAAGCGTGACCGATTATGTGCAGTGCGTGCTGAGCTGCCTGGGCGGCGGCCTGGCGCTGTGGCGTGCGGGCCAGTGGCTGTGCGCGCAGCGTCTGGGCCATTGCCATGTGTATTGGGCGATTGGCGAAGAACTGCTGCCGAGCTGCGGCCATAAACCGGATGGCGAAGTGCCGAAAGATCGTGAAGGCGGCGTGTTTAACCTGGGCCCGTTTATTACCCCGTGGCCGCCGGATCTGATTAACTTTACCGAAGGCAGCCGTCGTAGCCCGCTGTATACCCTGTGGTTTTGCGTGGGCCAGAGCTGGCCGCAGGATCAGCCGTGGATTAAACGTCTGGTGatgGTGAAAGTGCTGCCGCAGGTGGTGCCGatgTGCCTGCGTGTGCTGGTGAACATTGCGCGTCAGGGCGGCGCGAGCAGCCTGGAAAACACCGTGGATCTGCATATTAGCAACAGCCAGCCGCTGAGCCTGACCAGCGATCAGTATatgGCGTATCTGCAGGATCTGGCGGAAGATatgGATTTT

>Iran_Cashmere

atgGGCACCCAGAAACCGCGTATTCTGCCGTGGCTGATTAGCCAGCTGGATCGTGGCGAACTGGAAGGCGTGGCGTGGCTGGGCGAAAGCCGTACCCGTTTTCGTATTCCGTGGAAACATGGCCTGCGTCAGGATGCGCAGCAGGAAGATTTTGGCATTTTTCAGGTGCGTGCGAGCCGTGAAGGCGCGTGGGCGGAAGCGAGCGGCGCGTATACCCCGGGCAAAGATAAACCGGATCTGCCGACCTGGAAACGTAACTTTCGTAGCGCGCTGAACCGTAAAGAAGTGCTGCGTCTGGCGGAAGATCATAGCAAAGATAGCCAGGATCCGCATAAAATTTATGAATTTGTGAACAGCGGCGTGCTGGAAGTGGGCGTGCGTGATATTCCGGAAACCGATACCGCGCAGGATAACGGCCGTCATAGCACCAGCGATACCCAGGTGCGTCGTCTGCCGAGCCATGCGGCGTTTAGCGTGCAGGAAGATATTGTGCAGAAACTGCTGAGCGATatgGATCTGAGCCCGGAAGGCGGCCCGAGCAACCTGACCatgACCAGCGAAAACCCGCCGCAGCTGCTGCTGAGCCCGGAAAGCGATATTCCGGCGCTGTGCCCGAACAGCGGCCTGAGCGAAAACCCGCTGAAACAGCTGCTGGCGAACGAAGAAAGCGATTGGGAATTTGAAGTGACCGCGTTTTATCGTGGCTGCCAGGTGTTTCAGCAGACCGTGTTTTGCCCGGGCGGCCTGCGTCTGGTGGGCAGCGAAGCGGGCGATCGTatgCTGCCGGGCCAGCCGATTCGTCTGCCGGATCCGGCGGCGAGCCTGGCGGATAAAAGCGTGACCGATTATGTGCAGTGCGTGCTGAGCTGCCTGGGCGGCGGCCTGGCGCTGTGGCGTGCGGGCCAGTGGCTGTGCGCGCAGCGTCTGGGCCATTGCCATGTGTATTGGGCGATTGGCGAAGAACTGCTGCCGAGCTGCGGCCATAAACCGGATGGCGAAGTGCCGAAAGATCGTGAAGGCGGCGTGTTTAACCTGGGCCCGTTTATTACCCCGTGGCCGCCGGATCTGATTAACTTTACCGAAGGCAGCCGTCGTAGCCCGCTGTATACCCTGTGGTTTTGCGTGGGCCAGAGCTGGCCGCAGGATCAGCCGTGGATTAAACGTCTGGTGatgGTGAAAGTGCTGCCGCAGGTGGTGCCGatgTGCCTGCGTGTGCTGGTGAACATTGCGCGTCAGGGCGGCGCGAGCAGCCTGGAAAACACCGTGGATCTGCATATTAGCAACAGCCAGCCGCTGAGCCTGACCAGCGATCAGTATatgGCGTATCTGCAGGATCTGGCGGAAGATatgGATTTT

>Iran_Meat_goat

atgGGCACCCAGAAACCGCGTATTCTGCCGTGGCTGATTAGCCAGCTGGATCGTGGCGAACTGGAAGGCGTGGCGTGGCTGGGCGAAAGCCGTACCCGTTTTCGTATTCCGTGGAAACATGGCCTGCGTCAGGATGCGCAGCAGGAAGATTTTGGCATTTTTCAGGTGCGTGCGAGCCGTGAAGGCGCGTGGGCGGAAGCGAGCGGCGCGTATACCCCGGGCAAAGATAAACCGGATCTGCCGACCTGGAAACGTAACTTTCGTAGCGCGCTGAACCGTAAAGAAGTGCTGCGTCTGGCGGAAGATCATAGCAAAGATAGCCAGGATCCGCATAAAATTTATGAATTTGTGAACAGCGGCGTGCTGGAAGTGGGCGTGCGTGATATTCCGGAAACCGATACCGCGCAGGATAACGGCCGTCATAGCACCAGCGATACCCAGGTGCGTCGTCTGCCGAGCCATGCGGCGTTTAGCGTGCAGGAAGATATTGTGCAGAAACTGCTGAGCGATatgGATCTGAGCCCGGAAGGCGGCCCGAGCAACCTGACCatgACCAGCGAAAACCCGCCGCAGCTGCTGCTGAGCCCGGAAAGCGATATTCCGGCGCTGTGCCCGAACAGCGGCCTGAGCGAAAACCCGCTGAAACAGCTGCTGGCGAACGAAGAAAGCGATTGGGAATTTGAAGTGACCGCGTTTTATCGTGGCTGCCAGGTGTTTCAGCAGACCGTGTTTTGCCCGGGCGGCCTGCGTCTGGTGGGCAGCGAAGCGGGCGATCGTatgCTGCCGGGCCAGCCGATTCGTCTGCCGGATCCGGCGGCGAGCCTGGCGGATAAAAGCGTGACCGATTATGTGCAGTGCGTGCTGAGCTGCCTGGGCGGCGGCCTGGCGCTGTGGCGTGCGGGCCAGTGGCTGTGCGCGCAGCGTCTGGGCCATTGCCATGTGTATTGGGCGATTGGCGAAGAACTGCTGCCGAGCTGCGGCCATAAACCGGATGGCGAAGTGCCGAAAGATCGTGAAGGCGGCGTGTTTAACCTGGGCCCGTTTATTACCCCGTGGCCGCCGGATCTGATTAACTTTACCGAAGGCAGCCGTCGTAGCCCGCTGTATACCCTGTGGTTTTGCGTGGGCCAGAGCTGGCCGCAGGATCAGCCGTGGATTAAACGTCTGGTGatgGTGAAAGTGCTGCCGCAGGTGGTGCCGatgTGCCTGCGTGTGCTGGTGAACATTGCGCGTCAGGGCGGCGCGAGCAGCCTGGAAAACACCGTGGATCTGCATATTAGCAACAGCCAGCCGCTGAGCCTGACCAGCGATCAGTATatgGCGTATCTGCAGGATCTGGCGGAAGATatgGATTTT

>Capra_hircus

atgGGCACCCAGAAACCGCGTATTCTGCCGTGGCTGATTAGCCAGCTGGATCGTGGCGAACTGGAAGGCGTGGCGTGGCTGGGCGAAAGCCGTACCCGTTTTCGTATTCCGTGGAAACATGGCCTGCGTCAGGATGCGCAGCAGGAAGATTTTGGCATTTTTCAGGTGCGTGCGAGCCGTGAAGGCGCGTGGGCGGAAGCGAGCGGCGCGTATACCCCGGGCAAAGATAAACCGGATCTGCCGACCTGGAAACGTAACTTTCGTAGCGCGCTGAACCGTAAAGAAGTGCTGCGTCTGGCGGAAGATCATAGCAAAGATAGCCAGGATCCGCATAAAATTTATGAATTTGTGAACAGCGGCGTGCTGGAAGTGGGCGTGCGTGATATTCCGGAAACCGATACCGCGCAGGATAACGGCCGTCATAGCACCAGCGATACCCAGGTGCGTCGTCTGCCGAGCCATGCGGCGTTTAGCGTGCAGGAAGATATTGTGCAGAAACTGCTGAGCGATatgGATCTGAGCCCGGAAGGCGGCCCGAGCAACCTGACCatgACCAGCGAAAACCCGCCGCAGCTGCTGCTGAGCCCGGAAAGCGATATTCCGGCGCTGTGCCCGAACAGCGGCCTGAGCGAAAACCCGCTGAAACAGCTGCTGGCGAACGAAGAAAGCGATTGGGAATTTGAAGTGACCGCGTTTTATCGTGGCTGCCAGGTGTTTCAGCAGACCGTGTTTTGCCCGGGCGGCCTGCGTCTGGTGGGCAGCGAAGCGGGCGATCGTatgCTGCCGGGCCAGCCGATTCGTCTGCCGGATCCGGCGGCGAGCCTGGCGGATAAAAGCGTGACCGATTATGTGCAGTGCGTGCTGAGCTGCCTGGGCGGCGGCCTGGCGCTGTGGCGTGCGGGCCAGTGGCTGTGCGCGCAGCGTCTGGGCCATTGCCATGTGTATTGGGCGATTGGCGAAGAACTGCTGCCGAGCTGCGGCCATAAACCGGATGGCGAAGTGCCGAAAGATCGTGAAGGCGGCGTGTTTAACCTGGGCCCGTTTATTACCCCGTGGCCGCCGGATCTGATTAACTTTACCGAAGGCAGCCGTCGTAGCCCGCTGTATACCCTGTGGTTTTGCGTGGGCCAGAGCTGGCCGCAGGATCAGCCGTGGATTAAACGTCTGGTGatgGTGAAAGTGCTGCCGCAGGTGGTGCCGatgTGCCTGCGTGTGCTGGTGAACATTGCGCGTCAGGGCGGCGCGAGCAGCCTGGAAAACACCGTGGATCTGCATATTAGCAACAGCCAGCCGCTGAGCCTGACCAGCGATCAGTATatgGCGTATCTGCAGGATCTGGCGGAAGATatgGATTTT

>Nigerian_Red_Sokoto

atgGGCACCCAGAAACCGCGTATTCTGCCGTGGCTGATTAGCCAGCTGGATCGTGGCGAACTGGAAGGCGTGGCGTGGCTGGGCGAAAGCCGTACCCGTTTTCGTATTCCGTGGAAACATGGCCTGCGTCAGGATGCGCAGCAGGAAGATTTTGGCATTTTTCAGGTGCGTGCGAGCCGTGAAGGCGCGTGGGCGGAAGCGAGCGGCGCGTATACCCCGGGCAAAGATAAACCGGATCTGCCGACCTGGAAACGTAACTTTCGTAGCGCGCTGAACCGTAAAGAAGTGCTGCGTCTGGCGGAAGATCATAGCAAAGATAGCCAGGATCCGCATAAAATTTATGAATTTGTGAACAGCGGCGTGCTGGAAGTGGGCGTGCGTGATATTCCGGAAACCGATACCGCGCAGGATAACGGCCGTCATAGCACCAGCGATACCCAGGTGCGTCGTCTGCCGAGCCATGCGGCGTTTAGCGTGCAGGAAGATATTGTGCAGAAACTGCTGAGCGATatgGATCTGAGCCCGGAAGGCGGCCCGAGCAACCTGACCatgACCAGCGAAAACCCGCCGCAGCTGCTGCTGAGCCCGGAAAGCGATATTCCGGCGCTGTGCCCGAACAGCGGCCTGAGCGAAAACCCGCTGAAACAGCTGCTGGCGAACGAAGAAAGCGATTGGGAATTTGAAGTGACCGCGTTTTATCGTGGCTGCCAGGTGTTTCAGCAGACCGTGTTTTGCCCGGGCGGCCTGCGTCTGGTGGGCAGCGAAGCGGGCGATCGTatgCTGCCGGGCCAGCCGATTCGTCTGCCGGATCCGGCGGCGAGCCTGGCGGATAAAAGCGTGACCGATTATGTGCAGTGCGTGCTGAGCTGCCTGGGCGGCGGCCTGGCGCTGTGGCGTGCGGGCCAGTGGCTGTGCGCGCAGCGTCTGGGCCATTGCCATGTGTATTGGGCGATTGGCGAAGAACTGCTGCCGAGCTGCGGCCATAAACCGGATGGCGAAGTGCCGAAAGATCGTGAAGGCGGCGTGTTTAACCTGGGCCCGTTTATTACCCCGTGGCCGCCGGATCTGATTAACTTTACCGAAGGCAGCCGTCGTAGCCCGCTGTATACCCTGTGGTTTTGCGTGGGCCAGAGCTGGCCGCAGGATCAGCCGTGGATTAAACGTCTGGTGatgGTGAAAGTGCTGCCGCAGGTGGTGCCGatgTGCCTGCGTGTGCTGGTGAACATTGCGCGTCAGGGCGGCGCGAGCAGCCTGGAAAACACCGTGGATCTGCATATTAGCAACAGCCAGCCGCTGAGCCTGACCAGCGATCAGTATatgGCGTATCTGCAGGATCTGGCGGAAGATatgGATTTT

>Nigerian_WAD

atgGGCACCCAGAAACCGCGTATTCTGCCGTGGCTGATTAGCCAGCTGGATCGTGGCGAACTGGAAGGCGTGGCGTGGCTGGGCGAAAGCCGTACCCGTTTTCGTATTCCGTGGAAACATGGCCTGCGTCAGGATGCGCAGCAGGAAGATTTTGGCATTTTTCAGGTGCGTGCGAGCCGTGAAGGCGCGTGGGCGGAAGCGAGCGGCGCGTATACCCCGGGCAAAGATAAACCGGATCTGCCGACCTGGAAACGTAACTTTCGTAGCGCGCTGAACCGTAAAGAAGTGCTGCGTCTGGCGGAAGATCATAGCAAAGATAGCCAGGATCCGCATAAAATTTATGAATTTGTGAACAGCGGCGTGCTGGAAGTGGGCGTGCGTGATATTCCGGAAACCGATACCGCGCAGGATAACGGCCGTCATAGCACCAGCGATACCCAGGTGCGTCGTCTGCCGAGCCATGCGGCGTTTAGCGTGCAGGAAGATATTGTGCAGAAACTGCTGAGCGATatgGATCTGAGCCCGGAAGGCGGCCCGAGCAACCTGACCatgACCAGCGAAAACCCGCCGCAGCTGCTGCTGAGCCCGGAAAGCGATATTCCGGCGCTGTGCCCGAACAGCGGCCTGAGCGAAAACCCGCTGAAACAGCTGCTGGCGAACGAAGAAAGCGATTGGGAATTTGAAGTGACCGCGTTTTATCGTGGCTGCCAGGTGTTTCAGCAGACCGTGTTTTGCCCGGGCGGCCTGCGTCTGGTGGGCAGCGAAGCGGGCGATCGTatgCTGCCGGGCCAGCCGATTCGTCTGCCGGATCCGGCGGCGAGCCTGGCGGATAAAAGCGTGACCGATTATGTGCAGTGCGTGCTGAGCTGCCTGGGCGGCGGCCTGGCGCTGTGGCGTGCGGGCCAGTGGCTGTGCGCGCAGCGTCTGGGCCATTGCCATGTGTATTGGGCGATTGGCGAAGAACTGCTGCCGAGCTGCGGCCATAAACCGGATGGCGAAGTGCCGAAAGATCGTGAAGGCGGCGTGTTTAACCTGGGCCCGTTTATTACCCCGTGGCCGCCGGATCTGATTAACTTTACCGAAGGCAGCCGTCGTAGCCCGCTGTATACCCTGTGGTTTTGCGTGGGCCAGAGCTGGCCGCAGGATCAGCCGTGGATTAAACGTCTGGTGatgGTGAAAGTGCTGCCGCAGGTGGTGCCGatgTGCCTGCGTGTGCTGGTGAACATTGCGCGTCAGGGCGGCGCGAGCAGCCTGGAAAACACCGTGGATCTGCATATTAGCAACAGCCAGCCGCTGAGCCTGACCAGCGATCAGTATatgGCGTATCTGCAGGATCTGGCGGAAGATatgGATTTT

>Poitou

atgGGCACCCAGAAACCGCGTATTCTGCCGTGGCTGATTAGCCAGCTGGATCGTGGCGAACTGGAAGGCGTGGCGTGGCTGGGCGAAAGCCGTACCCGTTTTCGTATTCCGTGGAAACATGGCCTGCGTCAGGATGCGCAGCAGGAAGATTTTGGCATTTTTCAGGTGCGTGCGAGCCGTGAAGGCGCGTGGGCGGAAGCGAGCGGCGCGTATACCCCGGGCAAAGATAAACCGGATCTGCCGACCTGGAAACGTAACTTTCGTAGCGCGCTGAACCGTAAAGAAGTGCTGCGTCTGGCGGAAGATCATAGCAAAGATAGCCAGGATCCGCATAAAATTTATGAATTTGTGAACAGCGGCGTGCTGGAAGTGGGCGTGCGTGATATTCCGGAAACCGATACCGCGCAGGATAACGGCCGTCATAGCACCAGCGATACCCAGGTGCGTCGTCTGCCGAGCCATGCGGCGTTTAGCGTGCAGGAAGATATTGTGCAGAAACTGCTGAGCGATatgGATCTGAGCCCGGAAGGCGGCCCGAGCAACCTGACCatgACCAGCGAAAACCCGCCGCAGCTGCTGCTGAGCCCGGAAAGCGATATTCCGGCGCTGTGCCCGAACAGCGGCCTGAGCGAAAACCCGCTGAAACAGCTGCTGGCGAACGAAGAAAGCGATTGGGAATTTGAAGTGACCGCGTTTTATCGTGGCTGCCAGGTGTTTCAGCAGACCGTGTTTTGCCCGGGCGGCCTGCGTCTGGTGGGCAGCGAAGCGGGCGATCGTatgCTGCCGGGCCAGCCGATTCGTCTGCCGGATCCGGCGGCGAGCCTGGCGGATAAAAGCGTGACCGATTATGTGCAGTGCGTGCTGAGCTGCCTGGGCGGCGGCCTGGCGCTGTGGCGTGCGGGCCAGTGGCTGTGCGCGCAGCGTCTGGGCCATTGCCATGTGTATTGGGCGATTGGCGAAGAACTGCTGCCGAGCTGCGGCCATAAACCGGATGGCGAAGTGCCGAAAGATCGTGAAGGCGGCGTGTTTAACCTGGGCCCGTTTATTACCCCGTGGCCGCCGGATCTGATTACCTTTACCGAAGGCAGCCGTCGTAGCCCGCTGTATACCCTGTGGTTTTGCGTGGGCCAGAGCTGGCCGCAGGATCAGCCGTGGATTAAACGTCTGGTGatgGTGAAAGTGCTGCCGCAGGTGGTGCCGatgTGCCTGCGTGTGCTGGTGAACATTGCGCGTCAGGGCGGCGCGAGCAGCCTGGAAAACACCGTGGATCTGCATATTAGCAACAGCCAGCCGCTGAGCCTGACCAGCGATCAGTATatgGCGTATCTGCAGGATCTGGCGGAAGATatgGATTTT

>South_Africa_Goat

atgGGCACCCAGAAACCGCGTATTCTGCCGTGGCTGATTAGCCAGCTGGATCGTGGCGAACTGGAAGGCGTGGCGTGGCTGGGCGAAAGCCGTACCCGTTTTCGTATTCCGTGGAAACATGGCCTGCGTCAGGATGCGCAGCAGGAAGATTTTGGCATTTTTCAGGTGCGTGCGAGCCGTGAAGGCGCGTGGGCGGAAGCGAGCGGCGCGTATACCCCGGGCAAAGATAAACCGGATCTGCCGACCTGGAAACGTAACTTTCGTAGCGCGCTGAACCGTAAAGAAGTGCTGCGTCTGGCGGAAGATCATAGCAAAGATAGCCAGGATCCGCATAAAATTTATGAATTTGTGAACAGCGGCGTGCTGGAAGTGGGCGTGCGTGATATTCCGGAAACCGATACCGCGCAGGATAACGGCCGTCATAGCACCAGCGATACCCAGGTGCGTCGTCTGCCGAGCCATGCGGCGTTTAGCGTGCAGGAAGATATTGTGCAGAAACTGCTGAGCGATatgGATCTGAGCCCGGAAGGCGGCCCGAGCAACCTGACCatgACCAGCGAAAACCCGCCGCAGCTGCTGCTGAGCCCGGAAAGCGATATTCCGGCGCTGTGCCCGAACAGCGGCCTGAGCGAAAACCCGCTGAAACAGCTGCTGGCGAACGAAGAAAGCGATTGGGAATTTGAAGTGACCGCGTTTTATCGTGGCTGCCAGGTGTTTCAGCAGACCGTGTTTTGCCCGGGCGGCCTGCGTCTGGTGGGCAGCGAAGCGGGCGATCGTatgCTGCCGGGCCAGCCGATTCGTCTGCCGGATCCGGCGGCGAGCCTGGCGGATAAAAGCGTGACCGATTATGTGCAGTGCGTGCTGAGCTGCCTGGGCGGCGGCCTGGCGCTGTGGCGTGCGGGCCAGTGGCTGTGCGCGCAGCGTCTGGGCCATTGCCATGTGTATTGGGCGATTGGCGAAGAACTGCTGCCGAGCTGCGGCCATAAACCGGATGGCGAAGTGCCGAAAGATCGTGAAGGCGGCGTGTTTAACCTGGGCCCGTTTATTACCCCGTGGCCGCCGGATCTGATTAACTTTACCGAAGGCAGCCGTCGTAGCCCGCTGTATACCCTGTGGTTTTGCGTGGGCCAGAGCTGGCCGCAGGATCAGCCGTGGATTAAACGTCTGGTGatgGTGAAAGTGCTGCCGCAGGTGGTGCCGatgTGCCTGCGTGTGCTGGTGAACATTGCGCGTCAGGGCGGCGCGAGCAGCCTGGAAAACACCGTGGATCTGCATATTAGCAACAGCCAGCCGCTGAGCCTGACCAGCGATCAGTATatgGCGTATCTGCAGGATCTGGCGGAAGATatgGATTTT

>Ovis_aries

atgGGCACCCAGAAACCGCGTATTCTGCCGTGGCTGATTAGCCAGCTGGATCGTGGCGAACTGGAAGGCGTGGCGTGGCTGGGCGAAAGCCGTACCCGTTTTCGTATTCCGTGGAAACATGGCCTGCGTCAGGATGCGCAGCAGGAAGATTTTGGCATTTTTCAG---------------------GCGTGGGCGGAAGCGAGCGGCGCGTATACCCCGGGCAAAGATAAACCGGATCTGCCGACCTGGAAACGTAACTTTCGTAGCGCGCTGAACCGTAAAGAAGTGCTGCGTCTGGCGGAAGATCATAGCAAAGATAGCCAGGATCCGCATAAAATTTATGAATTTGTGAACAGCGGCGTGCGTGATATTCCG---------------GAACCGGATACCGCGCAGGATAACGGCCGTCATAGCACCAGCGAT---------------------------------------ACCCAGGAAGATATTGTGCAGAAACTGCTGAGCGATatgGATCTGAGCCCGGAAGGCGGCCCGAGCAACCTGACCatgACCAGCGAAAACCCGCCGCAGCTGCTGCTGAGCCGTGAAAGCGATATTCCGGCGCTGTGCCCGAACTGGGGCCTGAGCGAAAACCCGCTGAAACAGCTGCTGGCGAACGAAGAA---GATTGGGAATTTGAAGTGACCGCGTTTTATCGTGGCTGCCAGGTGTTTCAGCAGACCGTGTTTTGCCCGGGCGGCCTGCGTCTGGTGGGCAGCGAAGCGGGCGATCGTatgCTGCCGGGCCAGCCGATTCGTCTGCCGGATCCGGCGGCGAGCCTGACCGATAAAAGCGTGACCGATTATGTGCAGCGTGTGCTGAGCTGCCTGGGCGGCGGCCTGGCGCTGTGGCGTGCGGGCCAGTGGCTGTGCGCGCAGCGTCTGGGCCATTGCCATGTGTATTGGGCGATTGGCGAAGAACTGCTGCCGAGCTGCGGCCATAAACCGGATGGCGAAGTGCCGAAAGATCGTGAAGGCGGCGTGTTTAACCTGGGCCCGTTTATTACC------------GATCTGATTACCTTTACCGAAGGCAGCCGTCGTAGCCCGCTGTATACCCTGTGGTTTTGCGTGGGCCAGAGCTGGCCGCAGGATCAGCCGTGGATTAAACGTCTGGTGatgGTGAAA------------GTGGTGCCGatgTGCCTGCGTGTGCTGGTGGATATTGCGCGTCAGGGCGGCGCGAGCAGCCTGGAAAACACCGTGGATCTGCATATTAGCAACAGCCAGCCGCTGAGCCTGACCAGCGATCAGTATatgGCGTATCTGCAGGATCTGGCGGAAGATatgGATTTT
